# Everolimus affects vasculogenic mimicry in renal carcinoma resistant to sunitinib

**DOI:** 10.18632/oncotarget.9542

**Published:** 2016-05-21

**Authors:** Maria Serova, Annemilaï Tijeras-Raballand, Celia Dos Santos, Matthieu Martinet, Cindy Neuzillet, Alfred Lopez, Dianne C. Mitchell, Brad A. Bryan, Guillaume Gapihan, Anne Janin, Guilhem Bousquet, Maria Eugenia Riveiro, Ivan Bieche, Sandrine Faivre, Eric Raymond, Armand de Gramont

**Affiliations:** ^1^ AAREC Filia Research, Boulogne-Billancourt, France; ^2^ Department of Medical Oncology, Beaujon University Hospital (AP-HP - PRES Paris 7 Diderot), Clichy, France; ^3^ INSERM, Paris, France; ^4^ Department of Biomedical Sciences, Center of Emphasis in Cancer Research at The Paul Foster School of Medicine, Texas Tech University Health Sciences Center, El Paso, Texas, USA; ^5^ Department of Pathology Saint Louis University Hospital (AP-HP - PRES Paris 7 Diderot), Paris, France; ^6^ Department of Medical Oncology, Avicenne University Hospital (APHP- PRES Paris 13 University), Bobigny, France; ^7^ Laboratory of Oncogenetics, Institut Curie, Hôpital René Huguenin, St-Cloud, France

**Keywords:** everolimus, sunitinib, renal cell carcinoma, angiogenesis, differentiation

## Abstract

Angiogenesis is hallmark of clear cell renal cell carcinogenesis. Anti-angiogenic therapies have been successful in improving disease outcome; however, most patients treated with anti-angiogenic agents will eventually progress. In this study we report that clear cell renal cell carcinoma was associated with vasculogenic mimicry in both mice and human with tumor cells expressing endothelial markers in the vicinity of tumor vessels. We show that vasculogenic mimicry was efficiently targeted by sunitinib but eventually associated with tumor resistance and a more aggressive phenotype both *in vitro* and *in vivo*. Re-challenging these resistant tumors in mice, we showed that second-line treatment with everolimus particularly affected vasculogenic mimicry and tumor cell differentiation compared to sorafenib and axitinib. Finally, our results highlighted the phenotypic and genotypic changes at the tumor cell and microenvironment levels during sunitinib response and progression and the subsequent improvement second-line therapies bring to the current renal cell carcinoma treatment paradigm.

## INTRODUCTION

Clear cell renal cell carcinoma (ccRCC) is a highly metabolically deregulated cancer in which angiogenesis is a key aspect of pathogenesis, supporting the recent clinical successes of anti-angiogenic therapies in this disease [1]. Upregulation of angiogenesis is driven by stabilization of hypoxia-inducible transcription factors (HIFs) that are master regulators of the hypoxic response [2]. HIFs regulate several genes in the differentiation, angiogenesis, and metabolism pathways such as upregulation of the vascular endothelial growth factor (VEGF), a potent mediator of angiogenesis [3–6]. Besides cases in which HIF1α display very low expression due to 14q deletion, in many ccRCC, HIFs are constitutively stabilized through genetic inactivation of the von Hippel-Lindau (VHL) gene or mutations in TCEB1 (Elongin C), which impairs the function of the multimeric complex (VHL, Elongin B and C, Rbx1, Cul2) responsible for HIF ubiquitination [7, 8]. The resulting pseudo-hypoxic phenotype of ccRCC has been linked *in vitro* to constitutive upregulation of angiogenesis (VEGF), glucose metabolism (GLUT1, LDH-A, ALDOA, PGK1, PFKP), phosphate metabolism (AK-3), tissue remodeling (MMP1) and cell growth and differentiation (TGFB1, GMFB, IGFBP3) [9]. VHL inactivation has also been associated with genes whose regulation are not hypoxia-dependent, including genes involved in cell growth (CCND1, CDK6, IL- 6), cell migration and invasion (COL8A1, ITGB8) and immune regulation (CD59) [10]. However, in the context of VHL genetic inactivation and constitutive HIF activation, several VHL targets genes lose their hypoxia-inducible expression [11]. Nevertheless, despite putative different consequences between primary hypoxia (anti-angiogenic treatments in VHL wild-type tumors) and secondary hypoxia (anti-angiogenic treatments of VHL mutated tumors harboring a pre-existing pseudo-hypoxic phenotype), clinical benefits of anti-angiogenic therapies are not associated with VHL status [12–14].

In both RCC models and patients, treatment with the anti-angiogenic drug sunitinib has been shown to strikingly reduce tumor blood flow, which in turn induces cellular starvation, hypoxia, and necrosis [15–17]. However, most patients treated by sunitinib will eventually progress after a few months of therapy and switch for a second line therapy based on either mTOR inhibitors, such as everolimus, or on small TKI inhibitors such as sorafenib, axitinib or pazopanib [18–20]. Clinical and preclinical evidences have suggested that resistance to sunitinib is mediated through cancer cells and tumor microenvironment plasticity to adapt to a VEGFR-independent environment by activating other survival and angiogenic pathways such as PI3K/AKT/mTOR, IL-8 or FGF-2 pathways [21–25]. Resistance to sunitinib may also involved hypoxia-dependent mechanism such as vasculogenic mimicry by which tumor cells may acquire endothelial cell molecular markers and contribute to tumor perfusion [26]. In sunitinib resistant tumors, second line treatments with angiogenesis or mTOR inhibitors led to similar benefits [18]. However, recent clinical data suggested that second line drug efficacy may depend on the drug that primed the tumor for resistance. Whereas preliminary results from the SWITCH trial showed that there was no sequence effect using sunitinib and sorafenib, results from the RECORD-3 trial highlighted that everolimus treatment of sunitinib-resistant tumors was more efficacious than the reverse sequence; this later result suggests that sunitinib-dependent tumor adaptation is more specifically targetable by a mTOR inhibitor than are the everolimus-dependent tumor modifications by an anti-angiogenic agent [27]. Emerging evidence showed that both anti-angiogenic and anti-tumor activities of everolimus may be responsible for counteracting sunitinib resistance [28, 29].

Overall, phenotypic and genotypic changes at the tumor cell and microenvironment levels during sunitinib response and progression are poorly understood, as are the distinct effects of VEGFR-TKIs and mTOR inhibitors on these resistant tumors. A better understanding of these mechanisms may help improve the development of new compounds and rationalize the design of future clinical studies in ccRCC.

Herein, we report results from *in vitro* and *in vivo* RCC models with acquired resistance to sunitinib that were subsequently treated with everolimus, sorafenib or axitinib. We investigated the phenotypic and genotypic changes associated with sunitinib resistance with a particular focus on tumor cell differentiation and everolimus efficacy in re-challenging these resistant cells.

## RESULTS

### Responses of RCC tumors to first-line sunitinib

**Figure 1:**
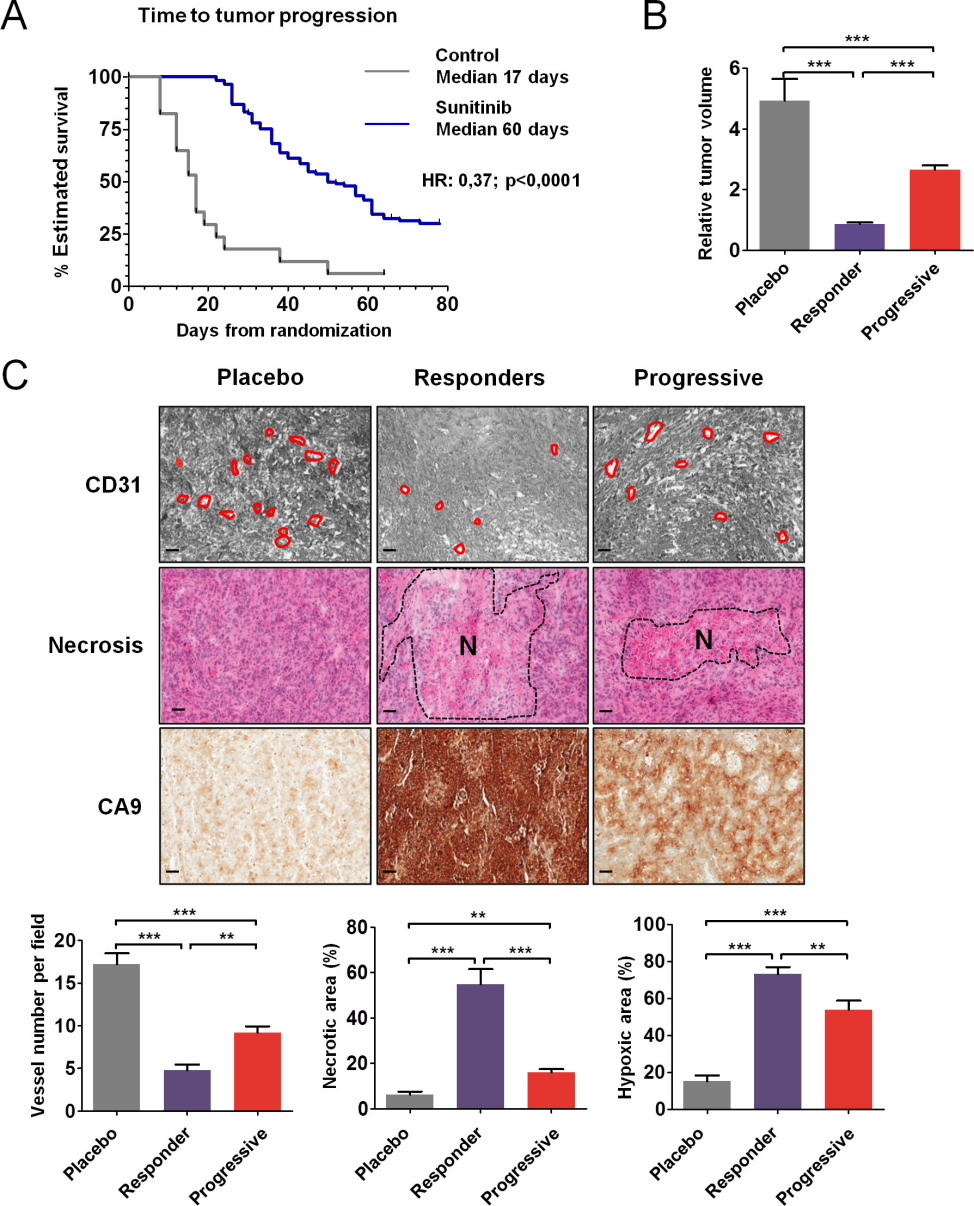
Antitumor and antiangiogenic effects of sunitinib in CAKI-1 RCC xenografts (**A**) Time to tumor progression (TTP) using Kaplan Meier estimate from 131 mice TTP data. (**B**) Relative tumor volume according to T0. Data are pooled from 15, 46 and 67 animals per group, respectively. Bars indicate the mean ± SEM. (**C**) Representative tumors from the placebo, responder, and progressive groups of vessel number, necrosis, and hypoxia detected by CD31, HE, and CA9 staining, respectively. The dashed region indicated the necrotic (N) area. Bars, 100 μm. (Below) Quantitative analysis of CD31, HE, and CA9 staining. Data are pooled from 5 animals per group. Bars indicate the mean ± SEM. (B and C) *P*-values were calculated using unpaired Student's *t* tests: **P < 0.01, ***P < 0.001.

CAKI-1 cells were subcutaneously injected into 131 nude mice (Figure S1). After 1 week, when tumors became palpable (50–100 mm^3^), the mice were randomly assigned to either vehicle control (*n* = 17) or sunitinib 60 mg/kg/day (*n* = 114). Median TTP was significantly longer in mice treated with sunitinib compared to placebo (50 days versus 17 days; HR = 0.37; 95% CI, 0.24–0.92; *P* < 0.0001) (Figure 1A). Tumor volumes were analyzed at the time of sacrifice for mice in the control and responder groups and before randomization for mice that progressed. Median relative tumor volume was decreased by 4.1-fold in responders compared to controls and median tumor size was increased by 2.4-fold in mice with progressive tumors compared to responders (*P* < 0.001) (Figure 1B). Mean tumor weight was significantly decreased in mice that responded to sunitinib treatment compared to controls (*P* < 0.001) and in responders compared to mice whose tumors progressed (*P* < 0.001); within each group, tumor volume correlated with tumor weight, which was measured after mice were sacrificed (data not shown). In clinical trials, sunitinib efficacy was shown to be independent of the VHL status of the tumors [12]. Alike, when engrafted 786–0 cells carrying a non functional VHL were treated by vehicle control or sunitinib 60 mg/kg/day, median TTP was significantly longer in mice treated with sunitinib compared to placebo (not reached versus 24 days; HR = 33.64; 95% CI, 4.94–229.2; *P* < 0.0002) (Figure S2A). Median relative tumor volume were significantly decreased in mice that responded to sunitinib treatment compared to controls (*P* < 0.001) and in responders compared to mice whose tumors progressed (*P* < 0.05) (Figure S2B).

### Vascularization and hypoxia in sensitive and resistant tumors to first line sunitinib

Analysis of representative tumors from each group of CAKI-1 mice showed that tumors from mice that responded to sunitinib were less vascularized than tumors from mice that progressed (Figure S1). Accordingly, CD31 staining revealed significantly fewer vessels in tumors from mice that responded to sunitinib, compared to controls (*P* < 0.001) (Figure 1C). In tumors from mice that progressed, the number of vessels was significantly greater than in responders (*P* < 0.01), but significantly fewer than in controls (*P* < 0.001). HE staining revealed more necrosis in tumors that were sensitive to sunitinib, both at the time of response (*P* < 0.001) and at the time of progression (*P* < 0.01), compared to untreated controls. The necrotic area was significantly greater in tumors that responded to sunitinib than in tumors that progressed (*P* < 0.001) (Figure 1C). CA9 staining revealed that the hypoxic area was increased in tumors that responded to sunitinib (*P* < 0.001) and in tumors that progressed (*P* < 0.05), compared to untreated controls (Figure 1C). Furthermore, areas of hypoxia were decreased in tumors that progressed, compared to tumors that responded to sunitinib treatment (*P* < 0.01). The distribution of CA9 staining was similar in control tumors and in tumors that progressed. In these tumors, hypoxia was concentrated at the rim between the tumor and the normal tissue, whereas in the tumors that responded to sunitinib treatment, the entire surface stained for CA9 and thus was hypoxic. Analysis of representative tumors from 786–0 mice showed that tumors from mice that responded or progressed under sunitinib displayed very similar phenotypes to tumors from CAKI-1 mice (Figure S2C). Tumor growth inhibition in response to sunitinib was associated with central necrosis, increased hypoxic area, and reduction of microvessel density. In tumors that progressed, necrosis, hypoxic area, and microvessel density reached intermediate values between control and responding tumors.

### *In vitro* response of sunitinib sensitive and resistant RCC cells to hypoxia

**Figure 2:**
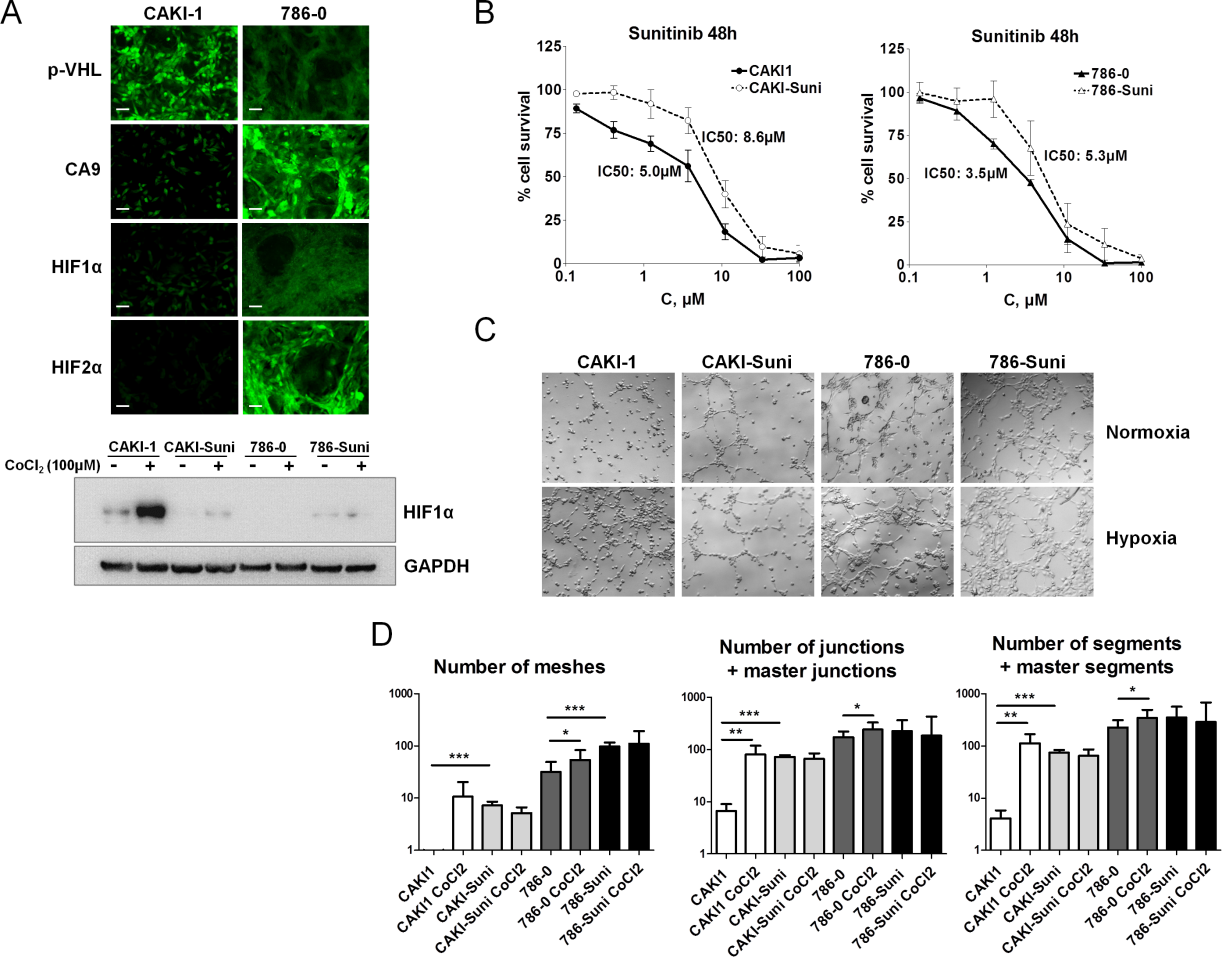
*In vitro* characterisation of acquired resistance to sunitinib in RCC cells (**A**) Expression of p-VHL, CA9, HIF1α and HIF2α by immunofluorescence in CAKI-1 and 786–0 cells under normoxia or hypoxia induced by 24 h exposure to 100 μM CoCl_2_. Representative pictures of three independent experiments. Bars, 100 μm. (**B**) Forty-eight hours sunitinib cytotoxicity in CAKI1, CAKI- Suni, 786–0 and 786-Suni models. (**C**) Pseudo-tubes formation by CAKI-1, CAKI-Suni, 786–0 and 786-Suni tumor cells on Matrigel after 24 h cell incubation in normoxia and hypoxia induced by 100 μM CoCl2. (**D**) Quantification of the tubes formation parameters by « Angiogenesis Analyzer » from Image J software. Data are pooled from three independent experiments. Bar indicate the mean ± SEM. *P*-values were calculated using unpaired Student's *t* tests: *P < 0.05, **P < 0.01, ***P < 0.001.

To investigate the role of hypoxia and drug resistance in the response of renal cancer cells to sunitinib, CAKI-1 cells carrying wild-type VHL and low basal expression of HIF-1α, HIF-2α and CA9 (Figure 2A), and 786–0 cells carrying mutated VHL, low basal HIF- 1α expression and high HIF-2α and CA9 protein levels were used to develop sunitinib resistant cell lines. For this purpose, cells were incubated in the presence of stepwise increased concentrations of sunitinib for more than 6 months. Resulting CAKI-Suni and 786-Suni cell lines were able to grow in the presence of higher concentrations of sunitinib up to 12 μM and displayed increased IC50 values of 8.6 μM and 5.3 μM, respectively, compared to the parental CAKI-1 and 786–0 cells (5.0 μM and 3.5 μM, respectively) (Figure 2B). Constitutive activation of AKT and ERK1/2 was observed in sunitinib resistant cells compared to their parental counterparts (Figure S3A and S3B). The effect of hypoxia was then evaluated in CAKI-1, 786–0 and sunitinib resistant cell lines. Cells were pretreated 24 hours with 100 μM CoCl_2_ to rapidly induce hypoxia in cell cultures. Hypoxia induction was confirmed by the dramatic increase of HIF-1α in CAKI- 1 cells whereas almost no changes were observed in CAKI- Suni, 786–0 and 786-Suni cell lines (Figure 2A). To evaluate the effects of hypoxia on vasculogenesis, we used a well-established *in vitro* model of pseudotube formation by renal cancer cells in hypoxic and normoxic conditions (see Materials and Methods). The numbers of meshes, junctions and segments were calculated for each experimental condition. In the CAKI- 1 cell line, we observed that the initially limited number of pseudotubes was increased after hypoxia pretreatment (Figure 2C and 2D). In contrast, untreated sunitinib resistant CAKI- Suni cells displayed a significantly higher number of pseudotubes than CAKI-1 cells that were not regulated by hypoxia. VHL-mutated 786–0 cells displayed a significantly higher capability for *in vitro* vasculogenesis compared to CAKI-1 or CAKI- Suni cells; in these cells, the number of pseudotubes was only slightly increased by hypoxia (Figure 2C and 2D). Sunitinib- resistant 786- Suni displayed a slightly higher number of pseudotubes than parental cells that were unchanged after hypoxia pretreatment. These data suggested that hypoxia can strongly induce vasculogenesis in CAKI-1 cells but significantly less in 786–0 cells that displayed high basal vasculogenesis capabilities. Acquired resistance to sunitinib in both CAKI-Suni and 786-Suni cells was associated with higher basal vasculogenesis capabilities compared to parental cells, but were no longer inducible by hypoxia. Therefore, exposure to hypoxia and sunitinib were both able to modify RCC cells phenotypes in a partially redundant manner.

### First-line sunitinib affects mouse and human-derived tumor angiogenesis

**Figure 3:**
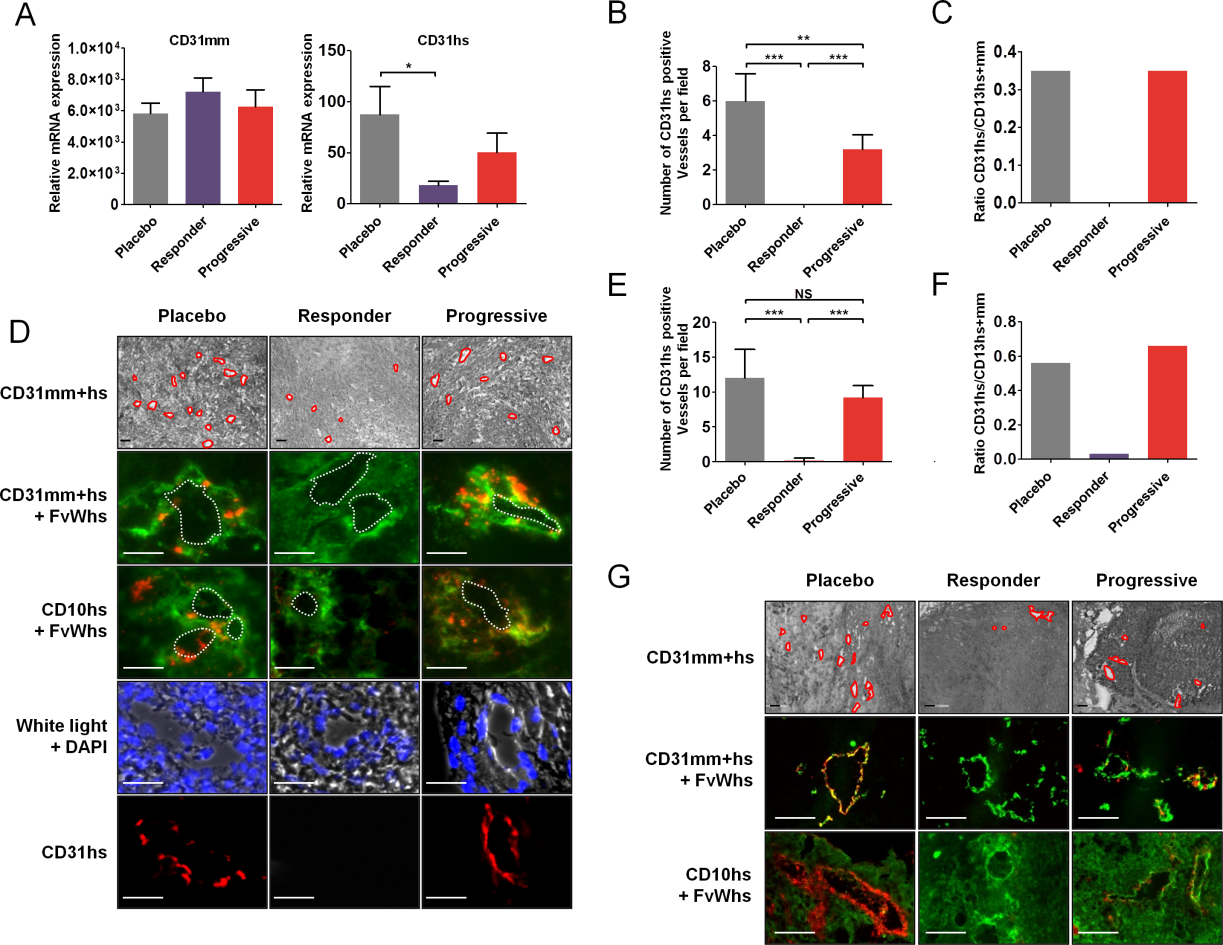
Tumor cells expressing endothelial cells markers in sunitinib first-line RCC xenografts (**A**) Quantification of CD31mm and CD31hs mRNA expression in placebo and sunitinib-treated tumors. Data are pooled from 6–8 animals per group. Bars indicate the mean ± SD. (**B**) Number of CD31hs-stained vessels per field from CAKI-1 xenografted tumors. Data are pooled from 6–8 animals per group. Bars indicate the mean ± SEM (**C**) Proportion of CD31hs positive vessels among CD31 positive vessels in CAKI- 1 xenografted tumors. Data are pooled from 6–8 animals per group. Bars indicate the ratio of the mean values. (**D**) Bright field and immunofluorescence examination of CD31 (CD31mm+hs), human CD31 (CD31hs), human FvW (FvWhs), and human CD10 (CD10hs) expression in placebo and sunitinib-treated tumors from CAKI-1 xenografts. Red lines indicate vascular lumens. Images are representative of 6–8 analysed mice. Bars, 100 μm. (**E**) Number of CD31hs-stained vessels per field from 786–0 xenografted tumors. Data are pooled from 6–8 animals per group. Bars indicate the mean ± SEM (**F**) Proportion of CD31hs positive vessels among CD31 positive vessels in CAKI- 1 xenografted tumors. Data are pooled from 6–8 animals per group. Bars indicate the ratio of the mean values. (**G**) Immunofluorescence analysis of CD31 (CD31mm+hs), human CD31 (CD31hs), human FvW (FvWhs), and human CD10 (CD10hs) expression in placebo and sunitinib-treated tumors from 786–0 xenografts. Images are representative of 6–8 analysed mice. Bars indicate the mean ± SEM. *P*-values were calculated using unpaired Student's *t* tests: *P < 0.05, **P < 0.01, ***P < 0.001, NS, *P* ≤ 0.05.

In mice, endothelial cells that form the tumor vascular network of engrafted tumor cells are from mouse origin. Since RCC cell lines displayed vasculogenesis capabilities *in vitro*, we evaluated whether these capabilities may be involved in tumor resistance *in vivo* through vasculogenic mimicry. By RT-PCR, there was no significant difference in relative expression of mouse CD31 (CD31mm) mRNA in tumors of mice receiving placebo or sunitinib in CAKI-1 tumors (Figure 3A). However, relative expression of human CD31 (CD31hs) mRNA was significantly lower in tumors of mice that responded to sunitinib, compared to control tumors (*P* < 0.05) (Figure 3A). Moreover, CD31hs expression was higher in tumors of mice that progressed under sunitinib than in tumors of mice that responded to sunitinib. Quantification of CD31hs staining by immunohistochemistry revealed that sunitinib affected the number of vessels associated with positive CD31hs cells at the time of response and progression. Tumors of mice that responded to sunitinib had significantly fewer vessels associated with positive CD31hs staining per field than tumors of mice that progressed (*P* < 0.001) (Figure 3B). Previous studies have shown that cancer cells may differentiate into endothelial cells and become involved in tumor angiogenesis by participating in vessel wall formation [30]. In placebo treated tumor cells, expression of human FvW (FvWhs) was observed in the vicinity of CAKI-1 tumor vessels as shown by FvWhs and CD31 co-staining (Figure 3D). Further suggesting that endothelial cells in the vicinity of vessel originate from human cancer cell, we found that FvWhs-stained cells coexpress human CD10 (CD10hs), a kidney-specific marker (Figure 3D). Interestingly, cells expressing FvWhs or CD31hs were detectable in the vicinity of tumor vessels, absent in tumors that responded to sunitinib treatment and present in the vessel rim of tumors at the time of progression (Figure 3D).

In 786–0 tumors, quantification of CD31hs staining revealed similar changes to CAKI-1 tumors, revealing a dramatic decrease of CD31hs positive vessels in tumors that responded to sunitinib compared to placebo or progressive tumors (Figure 3G). Similarly, co-staining of CD31 or CD10 with FvWhs showed that tumors cells expressing endothelial markers were detectable in 786– 0 tumors, absent in tumors that responded to sunitinib therapy and present at the time of tumor progression (Figure 3G). Of note, the proportion of vessels associated with positive CD31hs staining was higher in 786–0 tumors than in CAKI-1 tumors (56% vs 35%; Figure 3C and 3F) suggesting an increased differentiation and vasculogenesis potential of 786–0 cells.

### Tumoral CD31 staining in clear cell RCC patients

**Figure 4:**
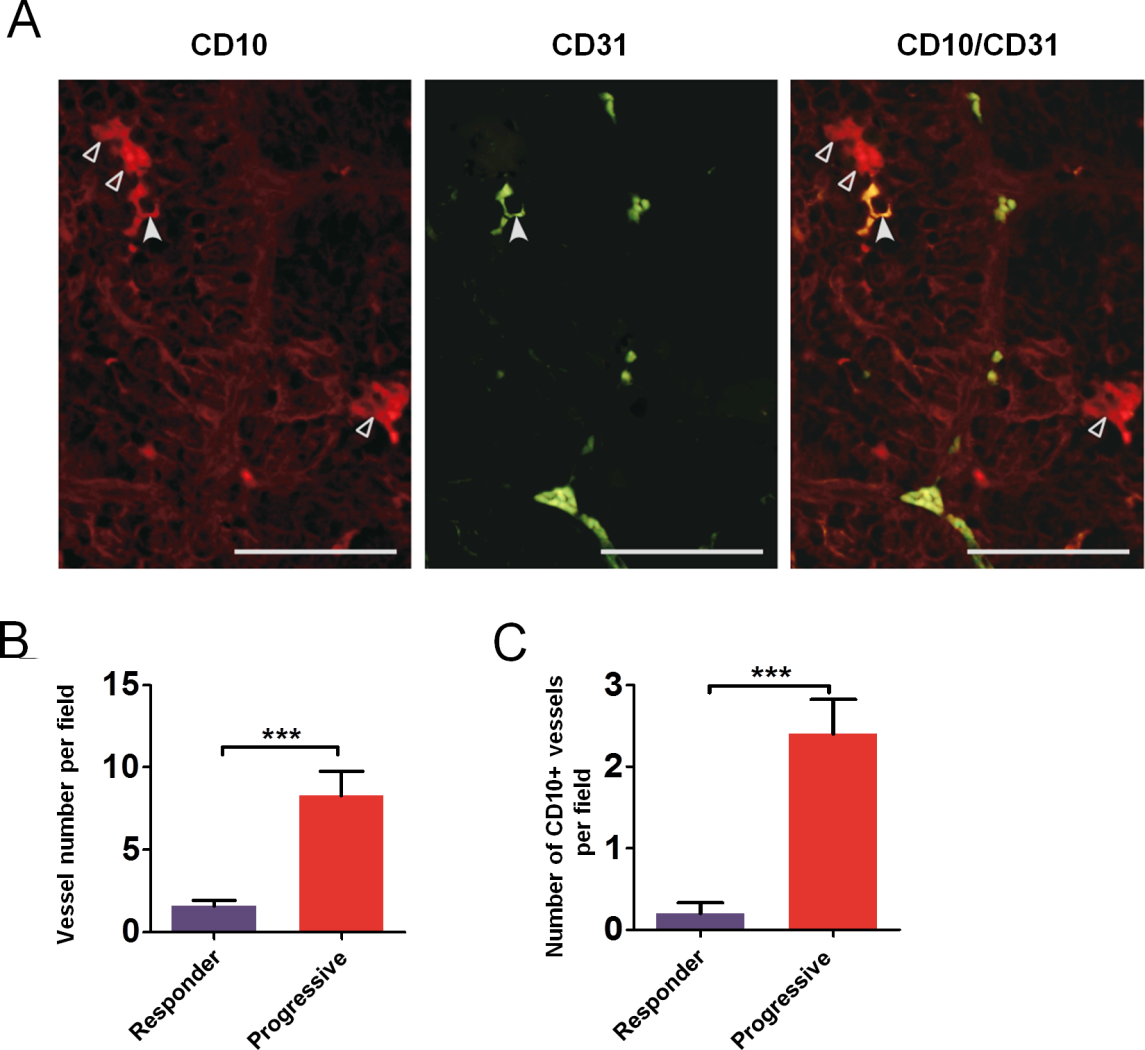
Tumor cells expressing endothelial cells markers in RCC patient samples Immunofluorescence examination of renal tumor cells stained with CD10 (hollow arrowheads, left) and vascular endothelial cells stained with CD31 (middle); one tumor cell was co-expressing CD10 and CD31 (plain arrowhead, right). Bars, 50 μm.

Tumors from a series of 24 patients were analyzed for expression of tumoral CD31 from biopsy samples or nephrectomy pieces. Patients' characteristics are summarized in Table S1. We performed CD31/CD10 costaining by immunofluorescence to identify CD31 expressing tumoral cells (Figure 4). CD31 staining was mainly membranous and focal. Among the 24 patients, 14 tumor samples (54%) displayed tumoral expression of CD31. When present, CD31 expressing tumor cells were localized in peri-vascular areas, mainly in clusters, in the vicinity of 3%–80% of the vessel (Table S2).

To investigate whether vasculogenic mimicry may occur in patients after treatment, we identified from our tissue bank two patients whose tumors were resected after treatment with sunitinib: one patient underwent tumor resection while experiencing a partial response and another patient underwent a removal of subcutaneous metastasis following tumor progression. Endothelial cells were readily detectable using FvWhs in renal tumors. Interestingly, CD10hs-stained renal cancer cells were detectable in the vicinity of vessels in tumor tissues suggesting that the phenotypic changes observed in mice models may also occur in some human samples.

### Effects of sunitinib on cancer cell differentiation

Looking at a panel of genes expression between sunitinib sensitive, or placebo, and sunitinib resistant models, we observed that variations in the mRNA expression of specific genes were different between *in vitro* and *in vivo* models, suggesting that tumor environment may affect the phenotype of tumor cells. As an example, whereas expression of E-cadherin (CDH1) was mainly unchanged between CAKI-1 and CAKI- Suni cells, *in vivo* sunitinib resistant tumors expressed significantly less CDH1 compared to placebo or sunitinib sensitive tumors (Figure S3B and S3C).

**Figure 5:**
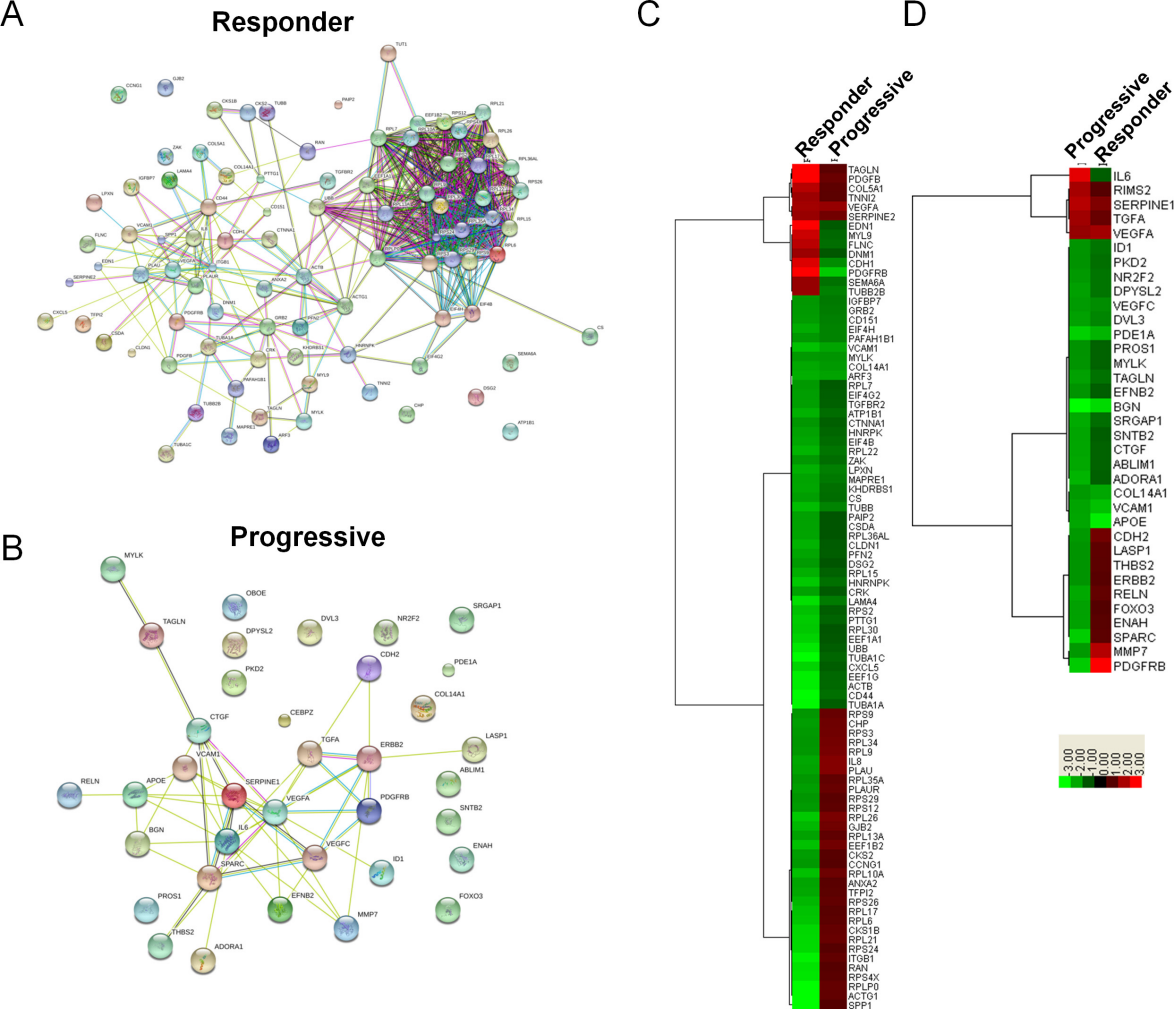
Expression analysis of differentially expressed genes identified in Metacore's top 10 process network maps for sunitinib progressive and responsive tumors (**A** and **B**) Functional Interaction Map for differentially expressed genes identified in Metacore's top 10 process network maps for sunitinib responsive (A) and progressive (B) tumors. All differentially expressed genes identified in Metacore's top 10 process network maps for sunitinib progressive tumors were input into the STRING functional interaction database. Lines between genes depict known functional interactions. (**C** and **D**) Hierarchical clustering of gene expression fold changes for the 90 genes and 35 genes identified in Metacore's top 10 process network maps for sunitinib responsive (C) and progressive (D) tumors.

**Figure 6:**
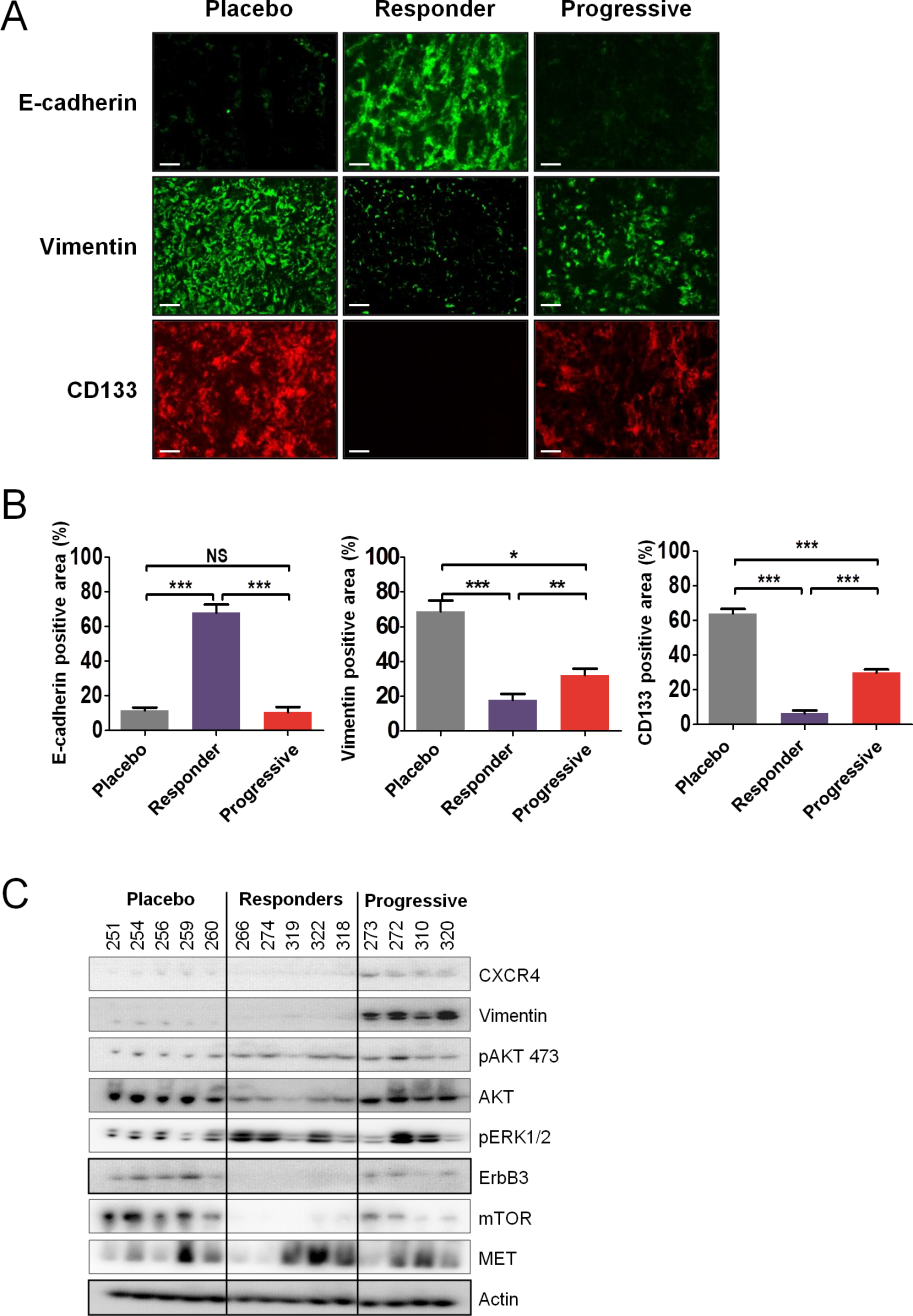
Sunitinib resistance is associated with tumor differentiation (**A** and **B**) Immunofluorescence examination (A) and quantitative analysis (B) of CD133, vimentin, and E-cadherin expression in placebo and sunitinib-treated tumors. Data are pooled from 6–8 animals per group. Bars indicate the mean ± SEM. *P*-values were calculated using unpaired Student's *t* tests: *P < 0.05, **P < 0.01, ***P < 0.001, NS, *P* ≤ 0.05. (**C**) Protein expression by western blot of selected genes in sunitinib-treated tumors. Representative expression of 4–5 mice per group.

To evaluate the underlying mechanisms modulating response to sunitinib *in vivo*, we collected placebo, sunitinib progressive, and sunitinib responding tumors from the hosts and subsequently examined their global transcription patterns using microarray technology. Relative to the placebo, statistically significant changes in gene expression (1.75-fold or greater; *p* ≤ 0.05) were observed for 925 genes (245 increased, 680 decreased) in the responders and 181 genes (21 increased, 160 decreased) in the progressive groups (Data File S1). As this is a large number of genes, we sought to identify the key clusters of genes that contribute to the response of RCC tumors to sunitinib. The lists of identified genes whose expression was greater than 1.75 fold in the responders and progressive groups were input into Metacore, an integrated software suite used for functional analysis of genomic data. Biological process network maps were generated, revealing that sunitinib responsive tumors display statistically significant differential gene expression patterns relative to the placebo reflective of tumor dependent growth and motility *i.e.* protein translation, cell adhesion and cytoskeletal dynamics, and cell cycle progression. The gene expression patterns of sunitinib progressive tumors relative to the placebo were characterized by statistically significant alterations in gene networks involved in the development of the tumor microenvironment *i.e.* angiogenesis and vascular morphogenesis, cell adhesion and cytoskeletal dynamics, cell guidance, and extracellular matrix remodeling. The process network identified with the highest significance for the progressive and responder groups is illustrated in Table 1. Of the top ten process networks that were identified by Metacore for the sunitinib responding and progressive tumor groups, we generated lists of the differentially expressed genes composing these networks, revealing 90 Metacore selected genes in the responder group and 35 genes in the progressive group (Figure 5). To identify the known direct and indirect interactions between the protein products of these genes, we input the lists of Metacore selected genes into the String database, revealing a remarkably strong node in the responders centered on a mass of genes involved in protein translation (Figure 5A), while interactions in progressive tumors demonstrated nodes involved in vascular regulation and cell adhesion (Figure 5B). These data strongly supported the biological process networks identified by Metacore. While these data indicate the varied responses of RCC tumors to sunitinib treatment, the majority of the gene expression changes in response to the drug were shared between the responding and progressive group. In order to identify the molecular distinction between the two groups, we generated hierarchical clustered heatmaps of the 90 responder and 35 progressive Metacore selected genes (Figure 5C and 5D) and focused our analysis on the most differentially regulated genes between each treatment. Based on the intensity values in the heatmaps, 51 genes were identified as Metacore selected differentially regulated transcripts. Of particular interest to our analysis, progressive and responder groups were characterized by differential expression of genes involved in cell adhesion and cytoskeletal dynamics (*ACTG1*_[P:N/C; R:↓]_*, SPARC*_[P:N/C; R:↓]_*, GJB2*_[P:N/C; R:↓]_, *CDH1*_[p:N/C; R:↑]_*, ITGB1*_[p:N/C; R:↑]_*, MYL9*_[p:N/C; R:↑]_*, TUBB2B*_[p:N/C; R:↑]_, and *MMP7*_[p:N/C; R:↑]_) and cell signaling (*PLAUR*_[P:N/C; R:↓]_*, PLAU*_[P:N/C; R:↓]_*, IL8*_[P:N/C; R:↓]_*, EDN1*_[p:N/C; R:↑]_*, ERBB2*_[P: ↓; R:N/C]_ *, FOXO3*_[P: ↓; R:N/C]_, and *PDGFRB*_[P: ↓; R: ↑]_). Several of these genes can also be considered as marker of tumor differentiation or contribute to epithelial-to-mesenchymal transition (EMT) such as CDH1, EDN1 or ERBB2 for instance. In fact, at the protein level, tumors of mice that progressed during sunitinib treatment expressed high levels of CD133 and vimentin compared to tumors of mice that responded to sunitinib (Figure 6A and 6B). In contrast, as in the previous gene expression analysis, strong E-cadherin expression was only observed in tumors of mice that responded to sunitinib. This suggested that while under strong hypoxia and reduced angiogenesis, effective sunitinib treatment affected genes and proteins involved in growth and differentiation in favor of a more epithelial phenotype than untreated tumors. In contrast, tumors of mice recovering from sunitinib eventually expressed genes involved in angiogenesis and differentiation, displaying an exacerbated mesenchymal phenotype in addition to the expression of endothelial genes.

**Table 1:** Top 10 most differentially regulated process for first and second line treatments in CAKI- 1 xenografts

Responders – sunitinib first line	
1	*Translation*_Translation initiation
2	*Translation*_Elongation-Termination
3	*Translation*_Elongation-Termination_test
4	*Cell adhesion*_Integrin-mediated cell-matrix adhesion
5	*Cytoskeleton*_Regulation of cytoskeleton rearrangement
6	*Cell cycle*_G2-M
7	*Cell cycle*_Mitosis
8	*Cell adhesion*_Platelet-endothelium-leucocyte interactions
9	*Cell adhesion*_Cell junctions
10	*Cytoskeleton*_Actin filaments
**Progressive – sunitinib first line**	
1	*Development*_Regulation of angiogenesis
2	*Cell adhesion*_Platelet-endothelium-leucocyte interactions
3	*Cell adhesion*_Attractive and repulsive receptors
4	*Development*_Neurogenesis_Synaptogenesis
5	*Development*_Neurogenesis_Axonal guidance
6	*Proteolysis*_ECM remodeling
7	*Cytoskeleton*_Actin filaments
8	*Development*_Blood vessel morphogenesis
9	*Apoptosis*_Anti-Apoptosis mediated by external signals via PI2K/AKT
10	*Signal transduction*_Androgen receptor signaling cross-talk
**Everolimus second line following sunitinib progression**	
1	*Translation*_Translation initiation
2	*Cell cycle*_Mitosis
3	*Protein folding*_response to unfolded proteins
4	*Cytoskeleton*_Regulation of cytoskeleton rearrangement
5	*Translation*_Elongation-Termination
6	*Translation*_Elongation-Termination_test
7	*Cell adhesion*_Cell junctions
8	*Protein folding*_Folding in normal condition
9	*Cell adhesion*_Integrin-mediated cell-matrix adhesion
10	*Cell adhesion*_Leukocyte chemotaxis
**Axitinib second line following sunitinib progression**	
1	*Translation*_Translation initiation
2	*Proteolysis*_Ubiquitin-proteasomal proteolysis
3	*Cell cycle*_Mitosis
4	*Translation*_Elongation-Termination
5	*Translation*_Elongation-Termination_test
6	*Transcription*_mRNA processing
7	*Immune response*_Antigen presentation
8	*Cytoskeleton*_Regulation of cytoskeleton rearrangement
9	*Cell cycle*_G2-M
10	*Immune response*_Phagosome in antigen presentation
**Sorafenib second line following sunitinib progression**	
1	*Translation*_Translation initiation
2	*Proteolysis*_Ubiquitin-proteasomal proteolysis
3	*Transcription*_mRNA processing
4	*Cell cycle*_Mitosis
5	*Translation*_Elongation-Termination
6	*Translation*_Elongation-Termination_test
7	*Cell cycle*_G1-S
8	*Cell cycle*_G2-M
9	*Cytoskeleton*_Regulation of cytoskeleton rearrangement
10	*Protein folding*_Folding in normal condition

All 1.75 fold or greater (*P* < 0.05) gene expression changes for first-line sunitinib responders and progressive tumors as well as second-line sorafenib, axitinib, or everolimus were input into Metacore software. The top 10 network process maps were identified using Metacore's proprietary algorithm and ranked according to statistical significance via *P* values.

### Responses of CAKI-1 tumors to second-line everolimus

**Figure 7:**
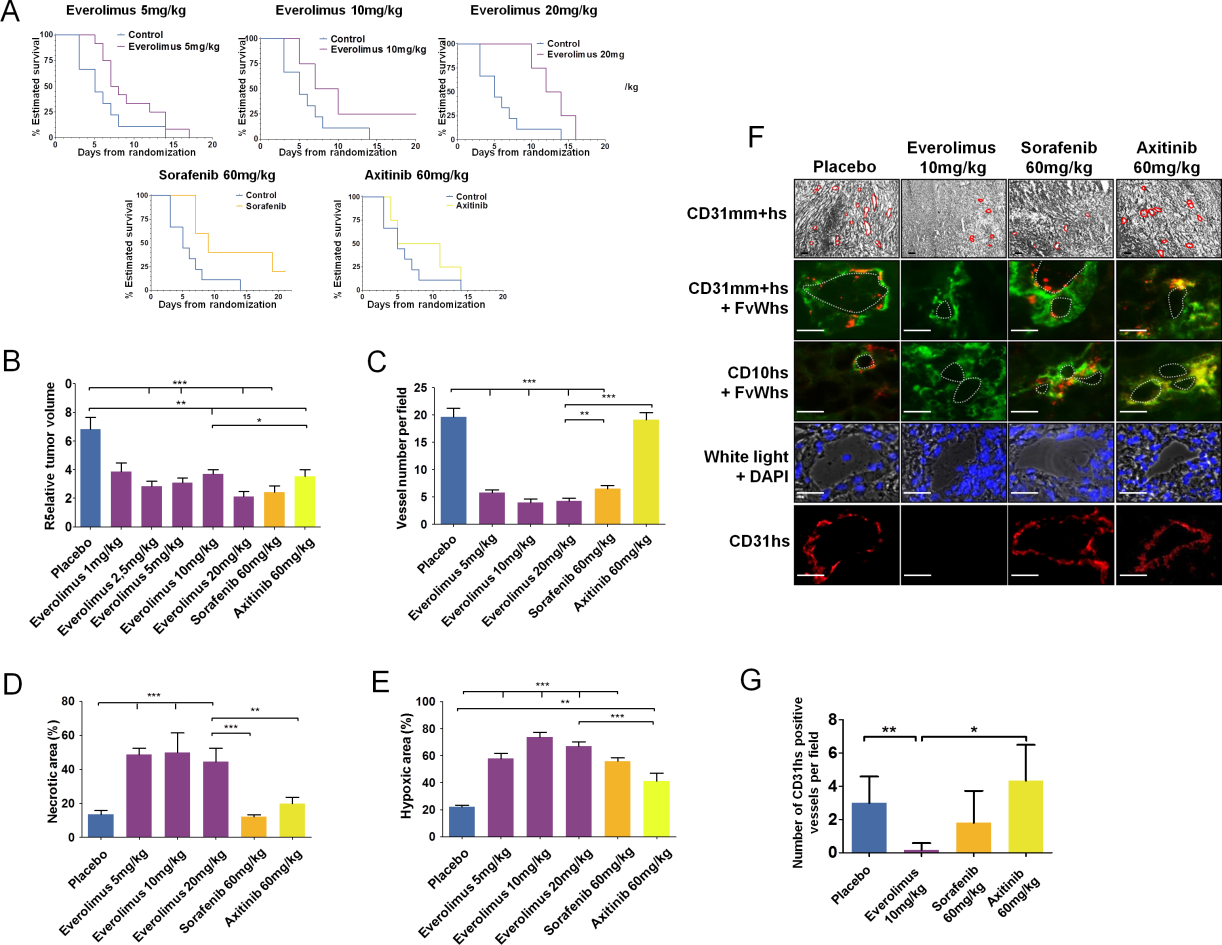
Everolimus effects on *in vivo* sunitinib-resistant tumors (**A**) Time to progression (TTP) of mice treated with everolimus (5, 10, and 20 mg/kg), sorafenib (60 mg/kg), or axitinib (60 mg/kg) using Kaplan Meier estimate from 56 mice TTP data. (**B**) Relative tumor volume at the end of second-line treatment with everolimus (1, 2.5, 5, 10, and 20 mg/kg), sorafenib (60 mg/kg), or axitinib (60 mg/kg) according to T0. Data are pooled from 4–12 animals per group. Bars indicate the mean ± SEM. (C-E) Vessel numbers per field (**C**), percentage of necrotic area (**D**) and hypoxic area (**E**) in sunitinib-resistant CAKI-1 tumors from mice treated with everolimus, sorafenib, or axitinib. Bars indicate the mean ± SEM. *P*-values were calculated using unpaired Student's *t* tests: *P < 0.05, **P < 0.01, ***P < 0.001. (**F**) Bright field and immunofluorescence examination of CD31 (CD31mm+hs), human CD31 (CD31hs), human FvW (FvWhs), and human CD10 (CD10hs) expression in tumors after second-line treatment with everolimus (10 mg/kg), sorafenib (60 mg/kg) or axitinib, (60 mg/kg). Red lines indicate vascular lumens. Bars, 100 μm. (**G**) Number of CD31hs stained vessels per field. Bars indicate the mean ± SEM of 10 high magnification fields. *P*-values were calculated using unpaired Student's *t* tests: *P < 0.05, **P < 0.01.

Two sets of experiments evaluating second-line treatment were performed. In the first set, CAKI-1 bearing mice that had progressed after sunitinib treatment received vehicle (*n* = 5), 1 mg/kg/day everolimus (*n* = 8), 2.5 mg/kg/day everolimus (*n* = 8), 5 mg/kg/day everolimus (*n* = 8), or 60 mg/kg/day sorafenib (*n* = 5). In the second set, mice received vehicle (*n* = 4), 5 mg/kg/day everolimus (*n* = 4), 10 mg/kg/day everolimus (*n* = 5), 20 mg/kg/day everolimus (*n* = 4), or 60 mg/kg/day axitinib (*n* = 5). No appreciable toxicity, defined by weight loss, unkempt appearance, mortality, and behavioral changes, was observed during treatment with everolimus, sorafenib, or axitinib. Median TTP with the highest dose of everolimus (20 mg/kg/day) was 13 days, which was significantly longer than the median TTP of 5 days observed in the control group (*P* < 0.001) (Figure 7A). Sorafenib and axitinib also showed prolonged TTP relative to control (non-significant differences because of small sample sizes). A significant decrease in tumor volume compared to placebo was observed in mice treated with all doses of everolimus (*P* < 0.001) (Figure 7B). Similar decreases in tumor volume were also seen with 60 mg/kg/day sorafenib and 60 mg/kg/day axitinib. A dose-dependent effect of everolimus was observed on tumor weight (data not shown). The reduction in tumor weight observed with 5–10 mg/kg/day everolimus was similar to those observed with 60 mg/kg/day sorafenib and 60 mg/kg/day axitinib.

### Effect of second line treatments on vascularization and hypoxia

CD31 staining of tumors from sunitinib-refractory CAKI-1-bearing mice treated with 5, 10, and 20 mg/kg/day everolimus (*n* = 5 for each group), sorafenib (*n* = 5), axitinib (*n* = 5), and vehicle control (*n* = 8) revealed that the number of vessels was significantly decreased in all everolimus- and sorafenib-treated tumors compared to control tumors (*P* < 0.001) (Figure 7C); everolimus 5 mg/kg/day induced an antiangiogenic effect similar to sorafenib treatment. No significant change in the number of vessels was observed in axitinib-treated tumors compared to control tumors. HE staining revealed a minimum of a 3-fold increase in necrotic areas in all everolimus-treated tumors compared to control tumors (*P* < 0.001) (Figure 7D). Neither sorafenib nor axitinib induced significant changes in necrotic surface area relative to control. No significant dose-dependent differences in necrosis were observed with everolimus. CA9 staining increased 5-fold in all everolimus- and sorafenib-treated tumors, compared to axitinib-treated and control tumors (Figure 7E). Everolimus effects on tumor hypoxia were not dose dependent, but sorafenib 60 mg/kg was significantly more effective at inducing hypoxia than axitinib 60 mg/kg (*P* < 0.001). In axitinib-treated and control tumors, CA9 staining was mainly confined to the tumor edges, whereas in the everolimus- and sorafenib-treated tumors, staining was evenly distributed.

### Second-line everolimus affects vasculogenic mimicry of sunitinib-resistant tumors

Immunohistochemical analysis demonstrated that sunitinib-resistant tumors of mice treated with everolimus expressed CD31mm, but not CD31hs, indicating that vessels contained endothelial cells of murine origin only (Figure 7F and 7G). Conversely, sunitinib-resistant tumors of mice treated with sorafenib or axitinib expressed CD31mm and CD31hs, highlighting the presence of endothelial cells with atypical phenotypes of murine and human origins. CD31 staining and CD31mm+hs/FvWhs co-staining revealed that sunitinib-resistant tumors treated with sorafenib or axitinib displayed vessels associated with an atypical endothelial cell phenotype in contrast to everolimus treated tumors (Figure 7F). These atypical tumoral/endothelial cells were shown to co-express CD10 and FvWhs confirming their human origin.

### Everolimus affects the differentiation of sunitinib-resistant cancer cells

**Figure 8:**
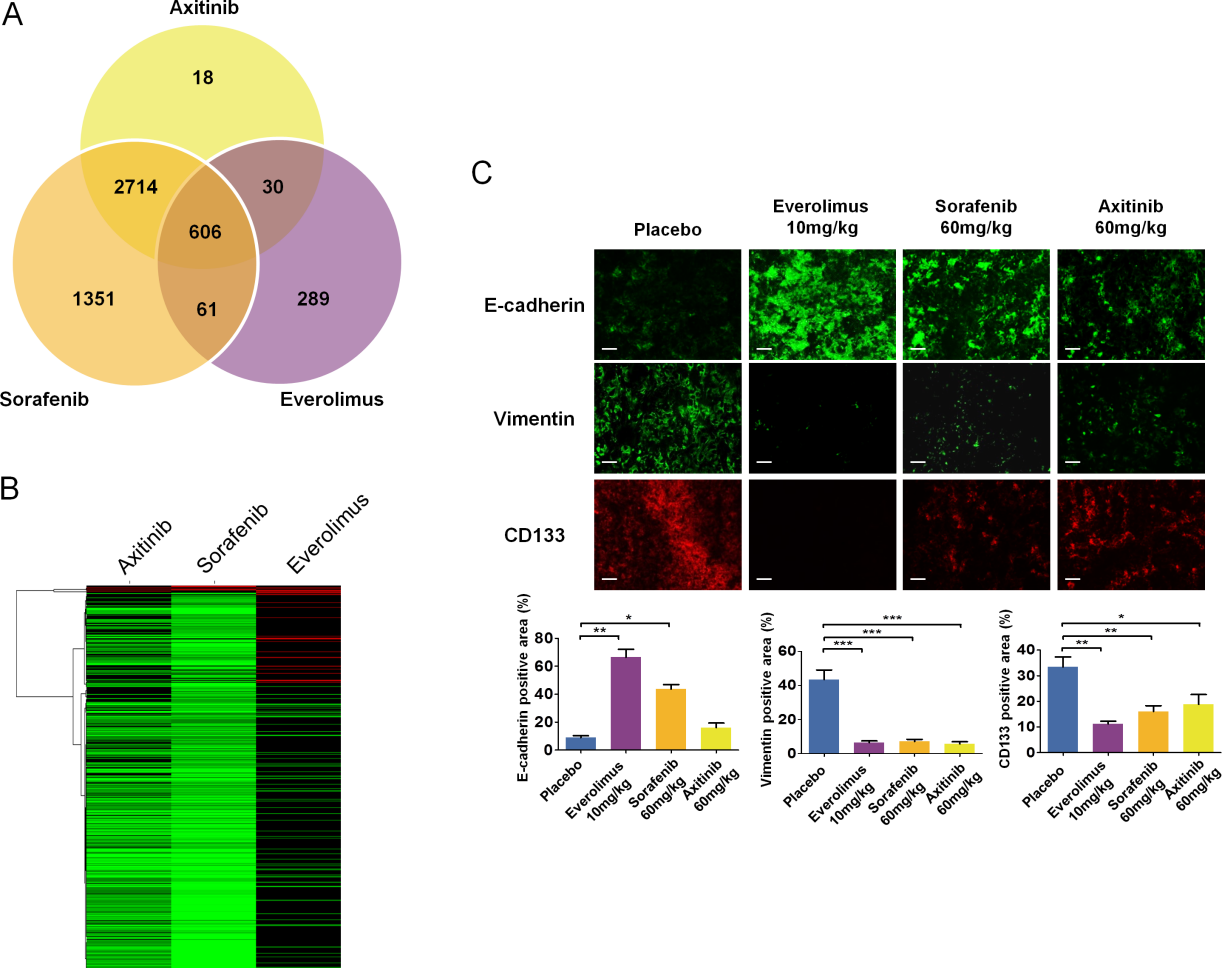
Molecular changes and tumor differentiation induced by second line treatment with everolimus, sorafenib and axitinib in sunitinib-resistant tumors (**A**) Identification of differentially regulated expression profiles in the second line treatments. All 1.75 fold or greater (*p* < 0.05) gene expression changes between the second line placebo and sorafenib, axitinib, or everolimus were input into a genomics Venn calculator to identify genes whose expression profiles were shared or unique between the three second line treatments relative to the placebo. (**B**) Hierarchical clustering of gene expression fold changes for the 372, 497, and 82 genes in the axitinib, sorafenib, and everolimus treatments, respectively. (**C**) Immunofluorescence examination and quantitative analysis of CD133, vimentin, and E-cadherin expression in tumors after second-line treatment with everolimus, axitinib, or sorafenib. Bars, 100 μm. Data are pooled from 4 animals per group. Bars indicate the mean ± SEM. *P*-values were calculated using unpaired Student's *t* tests: *P < 0.05, **P < 0.01, ***P < 0.001.

Given that axitinib, sorafenib, and everolimus altered the phenotypes of sunitinib resistant RCC tumors, we sought to gain an understanding of how these drugs differentially affected global gene expression patterns. Global microarray analysis was performed on second line axitinib, sorafenib, everolimus, and placebo treated tumors, revealing statistically significant expression changes (fold change ≥ 1.75, *p* ≤ 0.05) in 3835 (147 increased, 3688 decreased) genes in the axitinib treatment, 5314 (221 increased, 5093 decreased) genes in the sorafenib treatment, and 1057 (474 increased, 583 decreased) genes in the everolimus treated tumors relative to the placebo (Data File S2). Of these, 606 genes were similarly expressed in all three treatments compared to the control, while a number of genes were uniquely expressed in only one of the therapies (Figure 8A). All three drug treatment regimes were similarly characterized by process networks indicative of altered translation, cell cycle progression and cytoskeletal remodeling (Table 1). As we were surprised by the large number of down-regulated genes seen particularly in the axitinib and sorafenib treatments, the consistent expression changes seen in genes involved in protein translation likely accounts for a global down-regulation of gene expression as a whole. Given the observed phenotypic differences in the RCC tumors post-treatment with each drug, we sought to gain insight into how these drug treatments uniquely affected gene network processes in RCC tumors. To accomplish this, we input the list of genes identified as statistically significant into Metacore and compared the most differentially affected gene networks between the treatments, resulting in Metacore identification of 372 genes in the axitinib treatment, 497 genes in the sorafenib treatment, and 82 genes in the everolimus treatment. We generated heatmaps of these genes to identify the most differentially expressed genes between each treatment (Figure 8B). This heatmap revealed that axitinib and sorafenib were largely similar in expression profiles, with everolimus revealing the highest degree of differential expression between the three treatments. Our analysis revealed that the most differentially expressed genes in the everolimus treated tumors compared to the axitinib and sorafenib treated tumors consisted of genes involved in mRNA processing and protein translation (*UPF2, NOP2, UPF3A, NUPL2, CSTF3, HNRNPA2B1*), cytoskeletal regulation (*TUBB2B, MAPRE2, LMNB2*), angiogenesis (*PDGFA*), and miscellaneous (*HSP5A, FBXW7, MAL*). At the protein levels, tumors of mice treated with everolimus expressed low levels of CD133 and vimentin compared to tumors of control mice (Figure 8C). In contrast, strong E-cadherin expression was observed in tumors of mice treated with everolimus compared to tumors of control mice or treated with sorafenib or axitinib. Of note, expression of these proteins in sorafenib- and axitinib-treated tumors followed the trends of everolimus treatment although they were less pronounced. Downstream of mTOR inhibition, phosphorylation of S6 was lower in tumors of mice treated with all doses of everolimus and with axitinib than in control and sorafenib treated tumors (Figure S4A). ERK1/2 was activated in tumors of mice treated with everolimus, sorafenib, and axitinib, compared to control tumors. In contrast, while *in vitro* treatment of CAKI-1, 786–0 and their sunitinib resistant counterparts by everolimus affected mTOR signaling, it did not seem to affect the expression of genes such as vimentin (Figure S4B and S4C). These data confirm that *in vivo* and *in vitro* tumors respond differently to antitumor agents, which further emphasize the importance of the tumor microenvironment and demonstrate the impact of drug-resistant tumor models in preclinical studies.

## DISCUSSION

The VEGFR-TKI sunitinib is a recommended and commonly used first-line treatment for patients with metastatic ccRCC [31–33]. However, most patients with metastatic ccRCC who are treated with sunitinib will eventually develop resistance and subsequently experience disease progression. In this study, we developed *in vitro* and *in vivo* sunitinib resistant RCC models to investigate the phenotypic and genotypic changes associated with sunitinib resistance with a particular focus on tumor cell differentiation and everolimus efficacy in challenging resistant cells.

In RCC xenografts, sunitinib sensitive tumors, *i.e.* tumors whose growth was controlled by sunitinib, were associated with a reduction in micro-vessel density, increased hypoxia and central necrosis. These phenotypes were highly concordant with those observed in the clinic. In sunitinib resistant tumors, *i.e.* in tumors that progressed under sunitinib treatment, angiogenesis was restored increasing micro-vessel density while reducing hypoxia and necrosis. Interestingly, while models new vasculature typically arises from mouse stromal cells in xenograft, we found that mRNA and protein expression of human CD31 was higher in tumors that progressed during sunitinib treatment than in tumors that responded to sunitinib. As CD31 is an endothelial marker, this observation suggested that some endothelial cells may in fact originate from human cancer cells since they were the only source of human material transplanted into nude mice. To further investigate this phenomenon, we demonstrated that vessels from progressive tumors (control and sunitinib resistant tumors) displayed an atypical mixed phenotype with expression of human and mouse CD31 and human FvW (FvWhs). Co-staining of CD31 or CD10 with FvWhs revealed that these human endothelial markers were expressed by tumors cells and only in the tumor vessels' vicinity, which explained the atypical mixed phenotype of these tumor vessels. This finding is in line with results of a recent study, which demonstrated endothelial cell differentiation of glioblastoma stem-like cells and suggested that the ability of cancer stem-like cells to directly contribute to tumor vascularization through differentiation into endothelial cells may represent a new mechanism of angiogenesis [34]. This mechanism, called vasculogenic mimicry, might also represent incomplete differentiation of cancer stem-like cells toward endothelial lineage, as indicated by the atypical mixed phenotype of cells that retain the CD10 renal marker. Some authors also suggested that these mixed phenotypes could be the result of cell fusion instead of changes in cell differentiation fate [35]. Vasculogenic mimicry has been shown to play a role in tumor progression and has been detected in multiple tumor types, including breast, prostate, and ovarian carcinoma [36]. In a breast cancer xenograft model, vasculogenic mimicry was associated with neovascularization while being inducible by hypoxia and associated with expression of CD147 (matrix metalloproteinase inducer) in ovarian cancer cell models [37–39]. Our *in vitro* and *in vivo* results suggest that vasculogenic mimicry may exist in non-highly hypoxic RCC tumors as shown in *in vivo* control tumors that displayed ~30% of hypoxic areas. *In vitro*, the ability of RCC cells to form pseudotubes similar to that observed in endothelial HUVEC cells also suggests that besides hypoxia, vasculogenic mimicry may be induced or enhanced by sustained sunitinib exposure and VHL mutations. Although our clinical experience remains limited due to the complexity of obtaining serial samples at the time of progression in kidney cancer patients, our clinical data showed that CD31/CD10-stained renal cancer cells could be found in more than 50% of the primary tumors in patients presenting RCC, as well as in some patients who progressed under sunitinib therapy while being absent from patients responding to sunitinib. However this later observation should be further confirmed in the same patient tumor to rule out a difference that may come from a specific tumor background. Interestingly, given the absence of tumoral cells expressing endothelial markers in sunitinib responsive tumors, our results showed that sunitinib specifically affects this cell population whether it happens through changes in cancer cell differentiation or specific endothelial cell toxicity as suggested by some authors [40].

Our results showed that changes in gene expression upon exposure to sunitinib distinguished sensitive and resistant tumors. Compared to placebo, cancer cells in CAKI-1 xenografts sensitive to sunitinib displayed mRNA expression changes in pathways involved in tumor dependent growth and motility. In contrast, cancer cells resistant to sunitinib displayed gene expression modifications in pathways involved in the development of the tumor microenvironment. Considering that all tumors will eventually developed resistance to sunitinib, it is interesting to look at the gene expression changes between responders and progressive tumors as a chronological evolution of the same tumor. Given this perspective we can directly compare the gene expression changes between drug responsive and resistant tumors. Interestingly, progressive tumors re-expressed genes involved in translation and cell growth (TGF-α, CKS1B, CKS2, RAN), and while continuing expressing pro-angiogenic (PDGF-β, VEGFA) and pro-invasive factors (SERPINE1, SERPINE2, SPP1), increased expression of genes involved in cytoskeleton remodeling (PLAU, PLAUR, ACTG1) and inflammation (IL6, IL8) and decreased expression of genes involved in cell adhesion (CDH1); several of these genes having pleiotropic functions that may be involved in tumor progression. For some pathways, gene expression regulation between responder and progressive tumors may seem to be contradictory such as genes involved in cell adhesion; in fact, Gap junction beta-2 (GJB2) and Integrin beta-1 (ITGB1) genes were increased in progressive tumors whereas N-Cadherin (CDH2), SPARC and Metalloprotease 7 (MMP7) genes, which have been involved in promoting invasion, were decreased in responding tumors. Similarly, expression of the cell cycle inhibitor CCG1 was increased in progressive tumors. These apparently “contradictory” gene expression patterns may be the result of conditional gene function, *i.e.* genes that behave differently depending on tumor progression (genetic mutations in the gene pathway, changes in tumor environment) or global gene regulation due to gene methylation, which increases during tumor progression or tumor differentiation like the epithelial-to-mesenchymal transition (EMT). In fact, CCG1 has been shown to be increased to allow for EMT [41]. CAKI-1 tumors that progressed during sunitinib treatment also expressed more vimentin and less E-cadherin than tumors that responded to sunitinib treatment. This suggests that epithelial-mesenchymal transition (EMT), which is thought to be involved in tumor invasiveness and metastasis, is associated with sunitinib resistance. This observation is consistent with studies that demonstrated an association between EMT and sunitinib resistance in various tumor types including RCC [23, 30, 42]. It is interesting to note that changes in vimentin and E-cadherin were not apparent in our *in vitro* studies using sunitinib-resistant RCC cell lines. This suggests that EMT may be driven more by hypoxia and necrosis occurring in tumors than by direct pharmacological effects of sunitinib in cancer cells. This hypothesis is further supported by our observation that levels of the hypoxia-induced chemokine CXCL12 and its receptor CXCR4, which have been involved in tumor vascularization, invasion and lack of sunitinib efficacy [25, 43, 44], were increased in tumors that progressed, but not significantly altered in our *in vitro* experiments using sunitinib-resistant RCC cell lines. It would also be interesting to investigate specifically whether the atypical tumoral cells expressing CD31 express mesenchymal markers and display enhanced invasive capabilities since they may represent a tumor cell population particularly able to cross the vessel wall. Taken together, these results show that emergence of resistance to sunitinib during tumor progression may be associated with tumor cell differentiation and the emergence of an EMT-like phenotype in renal cell carcinomas.

Second-line therapies were investigated for their effects on tumor biology. Thereby, the number of mice in each group was not calculated to detect significant TTP differences between second-line treatment groups. It should also be noted that sorafenib and axitinib were each evaluated only at a single dose and that the relative exposures of these agents compared to each other and everolimus were not determined. Therefore, comparisons of TTP between these second-line therapies in this model must be made with caution. Gene expression analyses after second line treatment revealed that the main pathways involved in progressive tumor are similar among second line therapies affecting translation and cell cycle machineries as well as cytoskeletal rearrangement. Progressive tumors after everolimus treatment were slightly distinct from progressive tumors following sorafenib and axitinib treatments suggesting a different mode of action. We showed that second-line treatment with everolimus may affect mesenchymal cancer cell differentiation and angiogenesis recovery following acquisition of sunitinib resistance; tumors of mice treated with everolimus also had decreased vimentin and increased E-cadherin expression. Second-line treatment with everolimus in mice bearing sunitinib-resistant RCC xenografts slowed tumor progression, and inhibition of tumor growth was associated with increased necrosis and hypoxia and decreased micro-vessel density. It has been suggested that HIF-1α–induced hypoxia may activate MAPK signaling [45], which could explain the increased levels of p-ERK1/2 that we observed in everolimus-treated sunitinib-resistant tumors. Tumors of mice that progressed during sunitinib treatment and were subsequently treated with everolimus did not display human FvWhs staining. The absence of endothelial cells of human origin in mice treated with everolimus after progression to sunitinib demonstrates that everolimus might inhibit survival of cancer cell developing in the wall of blood vessels along with endothelial cell proliferation, mechanisms that both may play a role in the antiangiogenic effects of everolimus [46]. Sorafenib was associated with more hypoxic area in the tumors and induced fewer vessel numbers than axitinib. Unlike everolimus, sorafenib or axitinib did not decrease human FvWhs staining in vessels of sunitinib-resistant tumors. Our data suggest that tumors that are becoming resistant to sunitinib treatment may still be sensitive to alternative treatments such as everolimus that display multiple effects on tumor biology.

In summary, we demonstrated that tumors that progress during sunitinib treatment maintain a high level of vascularization suggesting that alternative proangiogenic pathways may be activated. We showed that tumor differentiation including vasculogenic mimicry, increased cooperation of the stroma, and combined autonomous pro-tumoral gene expression with a favorable tumor microenvironment may be necessary for tumor resistance to sunitinib. Everolimus slowed the progression of these sunitinib-resistant tumors, and tumor growth inhibition was associated with increased hypoxia and necrosis, decreased angiogenesis and a specific gene expression pattern compared to sorafenib and axitinib.

## MATERIALS AND METHODS

### Materials

Everolimus was supplied by Novartis. Sunitinib and axitinib were purchased from LC Laboratories (Woburn, MA, USA). For *in vivo* studies, sunitinib and axitinib powders were dissolved immediately before gavage in a sterilized solution containing 0.5% carboxymethylcellulose, 0.4% Tween 80, 1.8% NaCl, 0.9% benzyl alcohol for sunitinib and 0.5% carboxymethylcellulose for axitinib. Sorafenib (pharmacy department, Beaujon Hospital, Clichy, France) was available in its 200-mg tablet form and was dissolved fresh daily in Cremophor EL^®^/ethanol/H_2_O at 12.5%:12.5%:75% (Sigma, France). Everolimus microemulsion was suspended in sterile water at an appropriate concentration and was administered within 2 hours.

### Tumor cell xenografts in nude mice

All *in vivo* experiments were carried out with ethical committee approval and met the standards required by the United Kingdom Coordinating Committee on Cancer Research (UKCCCR) guidelines. CAKI-1 cells (5 × 10^6^) were injected subcutaneously into the flank of female athymic nude mice (Janvier, Le Genest St Isle, France) (Figure S1). Assuming that acquired sunitinib resistance will occur in ~50% of mice, approximately 133 mice should be randomized to expect 7 mice per second-line groups. One week after cell inoculation, all mice developed single subcutaneous palpable tumors of approximately 50 mm^3^ to 100 mm^3^. Mice were then randomly assigned to receive either 60 mg/kg/day sunitinib by oral gavage (5 days a week) or sterilized vehicle (0.5% carboxymethylcellulose, 0.4% Tween 80, 1.8% NaCl, 0.9% benzyl alcohol). The dose of sunitinib was chosen according to the literature [47] and to ensure the presence of two groups (responders and progressive after acquired resistance). Tumor volumes were measured 3 times per week along 2 major axes using calipers. Tumor volumes were calculated as follows: tumor volume = [(length) × (width^2^)]/2. Tumor progression was defined as 3 consecutive increases in tumor volume and a tumor volume that was double the initial tumor volume. These progressive tumors were considered to have developed acquired resistance to sunitinib. Within each group, tumor volume was correlated with tumor weight, which was measured after mice were killed. Progressive tumors under first-line sunitinib treatment were randomly assigned to receive second-line treatment with everolimus, sorafenib, or axitinib. Everolimus was administered at 5 doses (1, 2.5, 5, 10, and 20 mg/kg/day) by oral gavage (5 days per week for 3 weeks). The doses of everolimus used in this study were previously shown to be active in mouse xenografts. Sorafenib and axitinib, both dosed at 60 mg/kg/day, were used as reference treatments using the same administration protocol. Axitinib was shown to be active in a preclinical human colon cancer xenograft model at doses ranging from 30 to 100 mg/kg/day, and axitinib 30 mg/kg twice daily corresponded to an ED_70_ for inhibition of tumor growth and to the plasma IC_50_ for VEGFR-2 at 24 hours [48]. Sorafenib was used as described in the literature for CAKI-1 xenografts [49]. After 3 weeks or when the tumor volume reached 2 cm^3^, mice were sacrificed. Body weight and tumor weight were recorded. Time to progression (TTP), defined as the time from start of drug administration to tumor progression, was analyzed for first-line sunitinib treatment and for all second-line treatments.

### Cell lines

The human RCC cell lines CAKI-1 (VHL+/+) and 786–0 (VHL−/−) were obtained from the American Type Culture Collection (ATCC; Rockville, MD). CAKI-Suni and 786-Suni are variants of CAKI-1 and 786–0 exposed to sunitinib for more than 6 months. Cells were grown as monolayers in RPMI medium supplemented with 10% fetal calf serum (PAA, GE Healthcare Life Sciences, France), 2 mM glutamine, 100 units/mL penicillin, and 100 μg/mL streptomycin at 37°C in a humidified 5% CO_2_ atmosphere, and regularly checked for absence of *Mycoplasma*.

### Tissues samples from patients

Tissues samples were obtained from tumors stored in the Beaujon and St Louis Hospital Tissues Bank. Paraffin-embedded tissues were obtained from patients who provided informed consents for biological analysis of tumor tissues according to French National Guidelines.

### Immunohistochemistry and immunofluorescence of sunitinib-treated tumors

To characterize the mechanisms of acquired resistance to sunitinib, tumors from the control group, the sunitinib-responders group, and the sunitinib-resistant group were analyzed for necrosis, hypoxia, and angiogenesis. To characterize the effects of everolimus, sorafenib, and axitinib as second-line treatments, tumors from sunitinib-resistant mice treated with 5, 10, and 20 mg/kg everolimus, sorafenib 60 mg/kg, axitinib 60 mg/kg, and placebo were analyzed for necrosis, hypoxia, and angiogenesis. Immunohistochemistry was performed on OCT-embedded subcutaneous tumor stained with hematoxylin-eosin (HE) to evaluate necrosis, CA-IX (carbonic anhydrase 9, marker for downstream target of HIF-1α) to evaluate hypoxia, and CD31 (anti-human and anti-murine antibodies) to evaluate angiogenesis, using an automated immunohistochemical stainer. The images were captured and analyzed with a Zeiss Observer Z1 microscope. Quantifications were performed using Histolab software (Microvision, France). For the immunofluorescence assay, tumor slices were incubated with CD31, CD10, or von Willebrand factor (FvW) primary antibodies, followed by incubation with the secondary antibodies. Nuclei were stained with DAPI. Image analysis was performed using Zeiss Observer Z1 fluorescent microscope and AxioVision software. For the human samples, CD31/CD10 double immunofluorescence analyses were performed on 5-μm formalin-fixed paraffin-embedded tissue sections. Primary antibodies CD10 (clone: 56C6, 1:10, Novocastra, Leica, France) and CD31 (clone: JC70A, 1:4, Dako, France) were bound with Alexa 555 (APEX Alexa fluor^®^ 555 Antibody labeling kit, Invitrogen, France) and Alexa 488 (APEX Alexa fluor^®^ 488 Antibody labeling kit, Invitrogen, France) respectively. Double immunofluorescent staining was performed on a Discovery XT (Roche, France) by co-incubating tissue sections with the two bounded primary antibodies. Tissue sections were analyzed on a motorized Z-axis BX63 Olympus microscope (Rungis, France). Each fluorescent immunostaining was captured through a UPlan Fl/40 × /0.75 objective with a digital camera DP71 using Olympus CellSens Dimension software (Olympus, Rungis, France), using specific wavelength for fluorophore excitation. Merged pictures were performed to assess the co-localization of CD10 and CD31.

### mRNA gene expression

To evaluate and compare gene expression in the different tumor samples, RNA was extracted from 20 tumor slices from each first-line sunitinib treatment group (8 placebo, 6 sunitinib responders, and 6 sunitinib progressive) by RNABLE (Eurobio, France) standard protocol. Total RNA (1 μg) was reverse transcribed and the resulting cDNA were analyzed by quantitative real-time reverse transcriptase polymerase chain reaction (RT-PCR) for expression of selected target genes related to sunitinib resistance. mRNA expression of CXCR4, CXCL12, ErbB3, ErbB4, PDGFRA, PDGFRB, CDH1, and vimentin was analyzed in parental and sunitinib-resistant CAKI- 1 and 786–0 cells. Quantitative real- time RT-PCR was conducted through use of the ABI Prism 7900 Sequence Detection System (Perkin-Elmer Applied Biosystems, Foster City, CA, USA). Results were expressed as n-fold differences in target gene expression relative to the *TBP* gene (an endogenous RNA control) and relative to a calibrator (1× sample), consisting of the cell line sample from our tested series that contained the smallest amount of target gene mRNA. For microarray experiments, isolated RNA from each tumor was amplified, and biotin-labeled using Illumina TotalPrep RNA Amplification Kit (Illumina, San Diego, CA, USA). 750 ng of biotinylated aRNA was then briefly heat-denatured and loaded onto expression arrays to hybridize overnight. Following hybridization, arrays were labeled with Cy3-streptavidin and imaged using the Illumina ISCAN. Intensity values were transferred to Agilent GeneSpring GX microarray analysis software and data was filtered based on the quality of each call. Statistical relevance was determined using ANOVA with a Benjamini Hochberg FDR multiple testing correction (*p*-value < 0.05). Data were then limited by fold change analysis to statistically relevant data points demonstrating a 1.75-fold or greater alteration in expression of each gene when averaging the normalized values from each sample within an experimental group. Omics pathway analysis was performed with Metacore integrated software suite (www.portal.genego.com) and functional association networks were created using the String database (www.string-db.org).

### Protein expression

To evaluate and compare protein expression in the different tumor groups, total proteins were extracted from 25 to 30 tumor sections from each first-line sunitinib treatment group and analyzed for expression of CXCR4, vimentin, MET, ErbB3, AKT, p-AKT, p-ERK1/2, mTOR, p-S6, and actin by Western blot. Total proteins were also extracted from 25 to 30 tumor sections from each of the second-line treatment groups and analyzed for expression of p-S6, p-AKT, p-ERK1/2, vimentin, and actin by Western blot. Protein expression of p-Akt, p-ERK, Akt, PTEN, p27, and GAPDH in parental and sunitinib-resistant CAKI-1 and 786–0 cells and of p-S6, p-4EBP1, p-Akt, Akt, p-PKCα, p-ERK, vimentin, and GAPDH in cells exposed to 0.1 μM everolimus for 0, 1, 5, and 24 hours was assessed using Western Blot analysis.

### Cell viability

Parental and sunitinib-resistant CAKI-1 and 786–0 cells were exposed to 1 μM, 5 μM, 10 μM, and 20 μM everolimus and assessed for inhibition of cellular proliferation after 24–, 48–, 72–, and 96-hour exposure, using the MTT assay (3-[4x,5-dimethylthiazol-2-yl]-2,5-diphenyltetrazolium bromide; Sigma, Saint-Quentin Fallavier, France). In brief, cells were seeded at a density of 2 × 10^3^ cells/well. After 48 hours of incubation with sunitinib and 24 hours of postincubation in drug-free medium, cells were incubated with 0.4 mg/mL MTT. After incubation, formazan precipitates were dissolved and absorbance was measured at 560 nm (Thermo, France). Wells with untreated cells or with drug-containing medium without cells were used as positive and negative controls, respectively. Growth inhibition curves were plotted as the percentage of untreated control cells.

### *In vitro* angiogenesis assay

Well-established *in vitro* model of pseudotube formation by renal cancer cells was used to assess the *in vitro* angiogenesis in hypoxic and normoxic conditions. Briefly, renal cancer cells after 24 hour hypoxia (100 μM CoCl_2_) or normoxia incubation were plated on Matrigel-coated μ-Slides Angiogenesis (Ibidi, Biovalley, France) at concentration of 7 × 10^3^ per well. Pseudotube formation after 4 hours was visualized using phase contrast microscopy. The architecture of the matrix-associated vascular channels *in vitro*, which was characterized by interconnected loops and networks, modelled the network patterns observed in cultures by endothelial cells. Number of meshes, junctions and segments was calculated using the “Angiogenesis Analyzer” software from Image J software developed by J. Carpentier (Creteil, France) (Figure S5).

### Statistics

All statistical analyses were performed using GraphPad Prism, Version 5.00, GraphPad Software. A nonlinear regression curve fit (1-phase exponential decay) was used to analyze dose response experiments. Two-tailed unpaired *t* test was used to calculate the significance of differences between groups (NS, not significant; *P < 0.05; **P < 0.01; and ***P < 0.001). Kaplan-Meier curves were constructed for survival analysis.

## SUPPLEMENTARY MATERIALS FIGURES AND TABLES, DATAS






