# Supplementary material for: Everolimus affects vasculogenic mimicry in renal carcinoma resistant to sunitinib
Source: Oncotarget. 2016 May 21;7(25):38467–86. doi: 10.18632/oncotarget.9542 (PMC5122404; doi:10.18632/oncotarget.9542)
Supplement: Supplementary file 1 [file oncotarget-07-38467-s001.pdf]

Everolimus affects vasculogenic mimicry in renal carcinoma resistant to sunitinib

Supplementary Materials

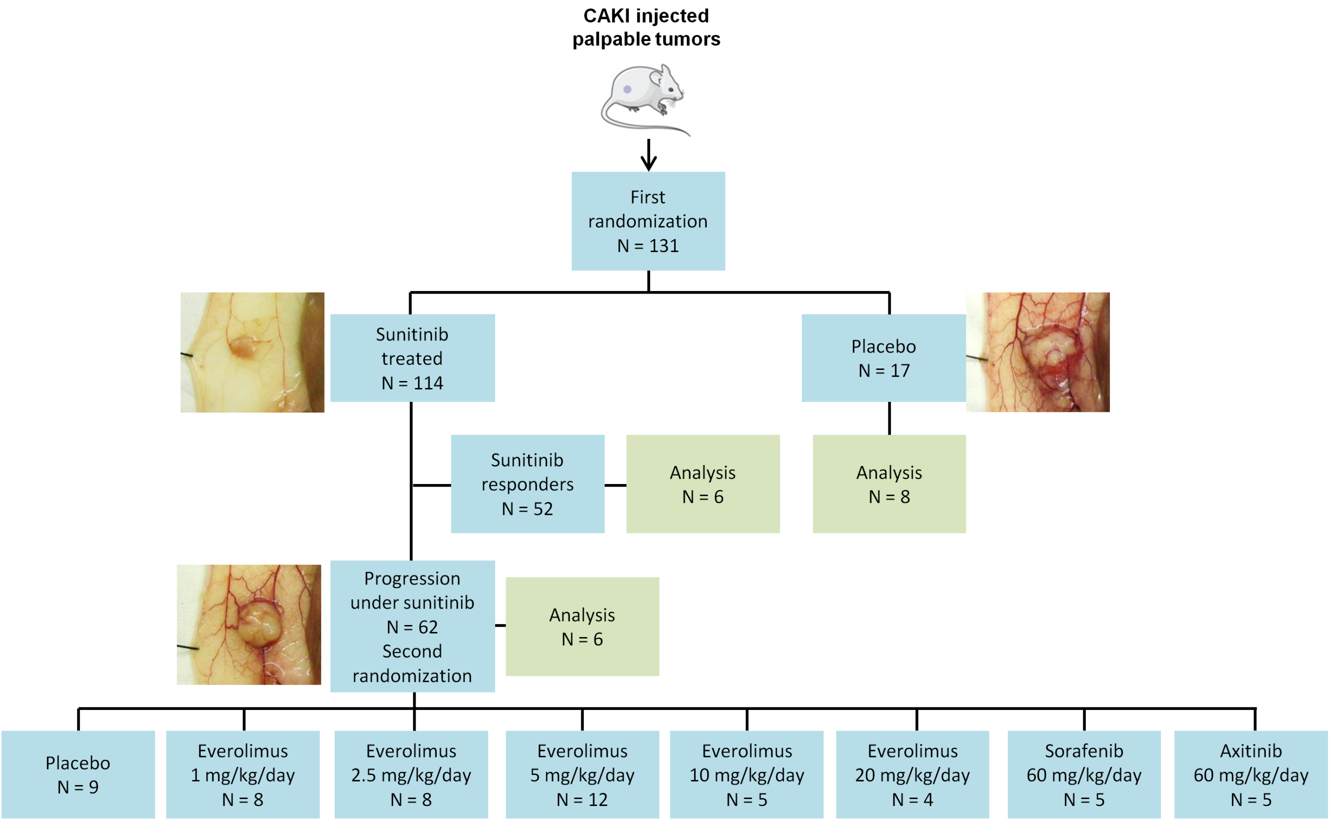

Supplementary Figure S1: *In vivo* study design in CAKI-1 xenografts and representative pictures of tumors from mice receiving placebo or responding and progressing under sunitinib treatment.

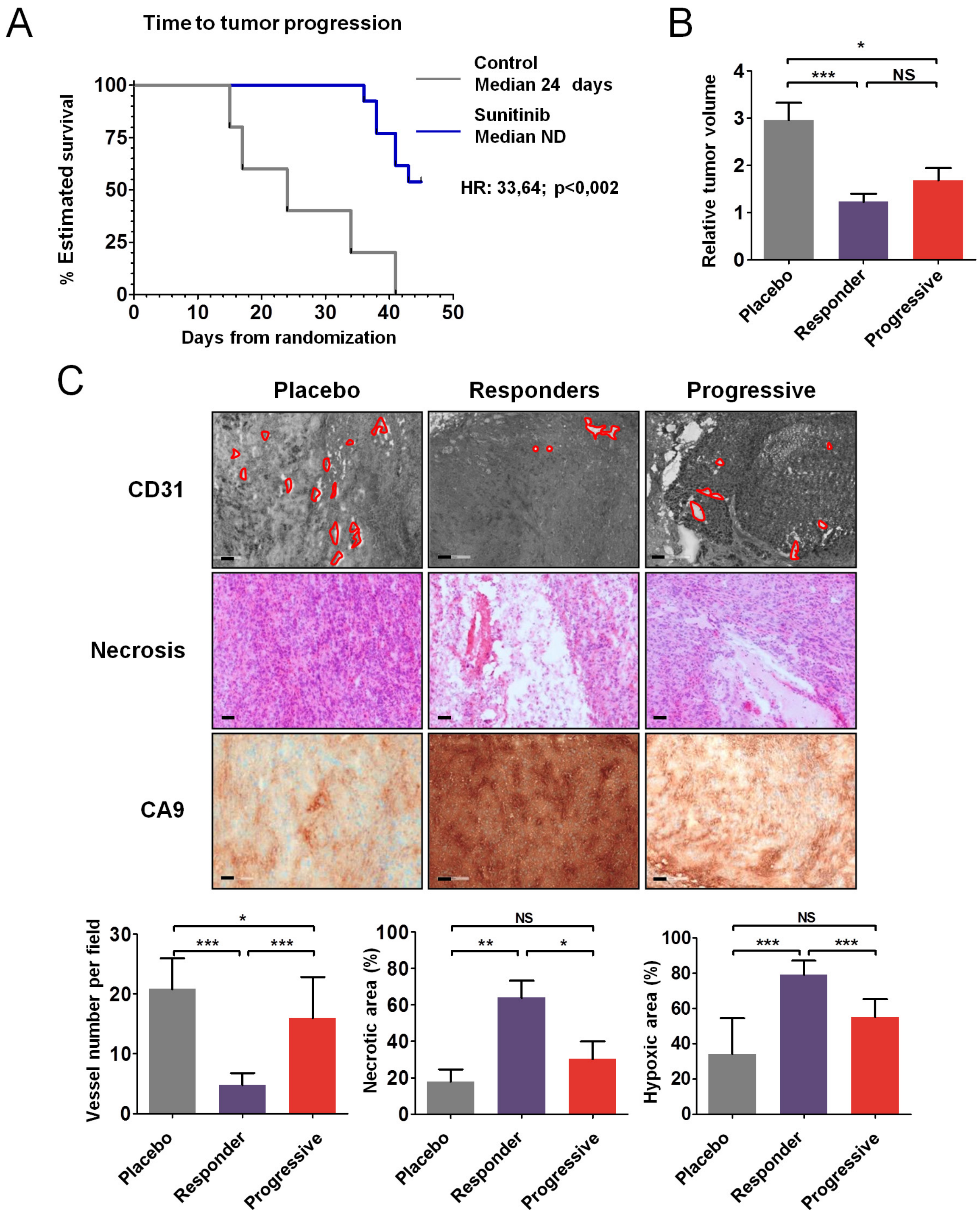

Supplementary Figure S2: Antitumor and antiangiogenic effects of sunitinib in 786–0 RCC xenografts. (**A**) Time to tumor progression (TTP) using Kaplan Meier estimate from X mice TTP data. (**B**) Relative tumor volume according to T0. Data are pooled from 5–7 animals per group. Bars indicate the mean ± SEM. (**C**) Representative tumors from the placebo, responder, and progressive groups and analysis of vessel number, necrosis, and hypoxia detected by CD31, HE, and CA9 staining, respectively. The dashed region indicated the necrotic (N) area. Bars, 100 µm. (Below) Quantitative analysis of CD31, HE, and CA9 staining. Data are pooled from 4–7 animals per group. Bars indicate the mean ± SEM. (B and C) *P*-values were calculated using unpaired Student's *t* tests: $*P < 0.05$, $**P < 0.01$, $***P < 0.001$, NS, $P < 0.05$.

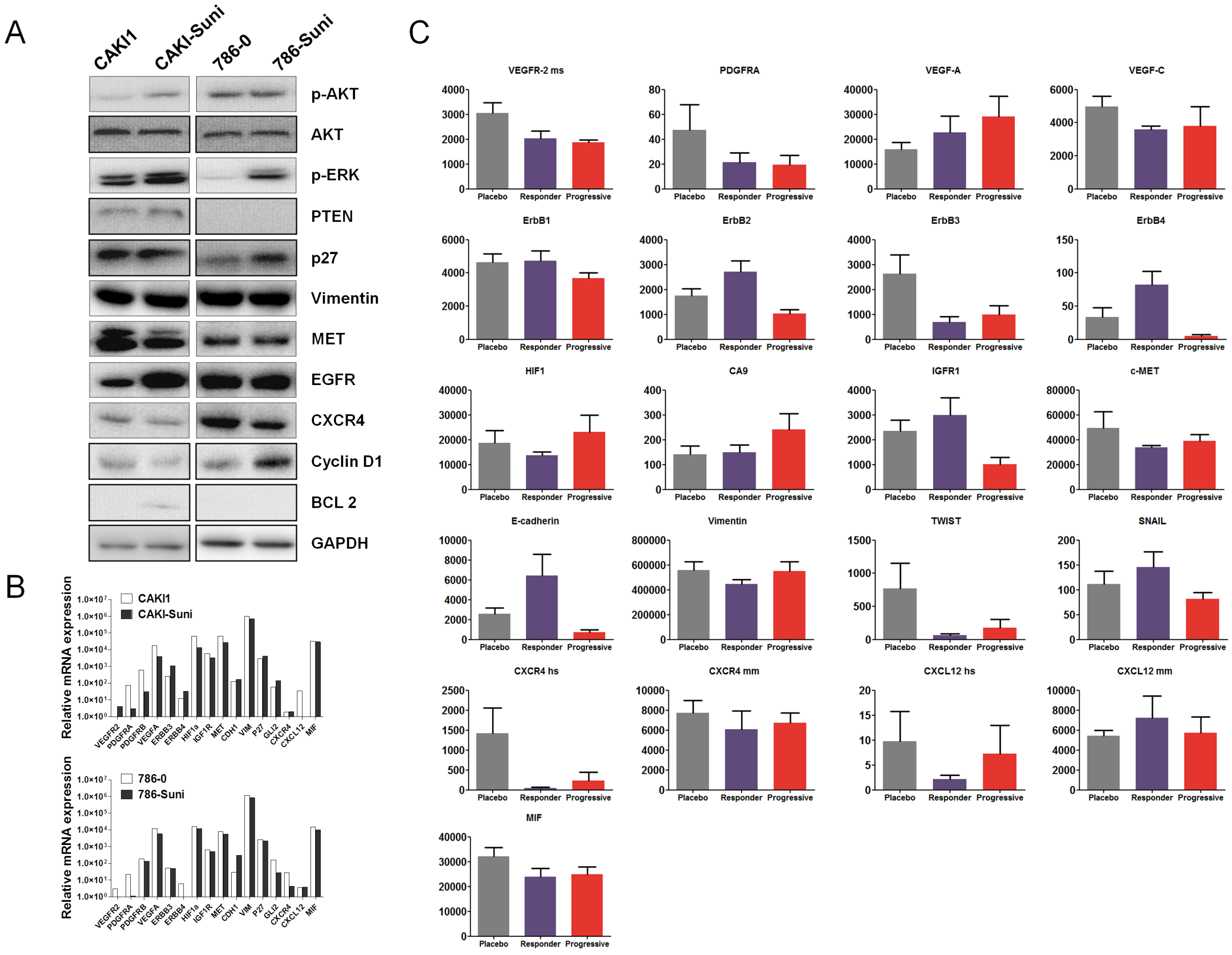

Supplementary Figure S3: *In vitro* characterisation of acquired resistance to sunitinib in RCC cells. (**A**) Protein expression by western blot of p-AKT, p-ERK1/2, AKT, PTEN, p27, Vimentin, MET, EGFR, CXCR4, Cyclin D1, BCL-2 and GAPDH in parental and resistant cell lines. (**B** and **C**) Quantitative analysis of selected gene expression using qRT-PCR in CAKI1, CAKI-Suni, 786-0 and 786-Suni cell lines (B) and in tumors from the placebo, and sunitinib-treated groups from CAKI-1 xenografted tumors (C). Bars indicate the mean ± SEM of three independent experiments or data pooled from 6–8 animals per group.

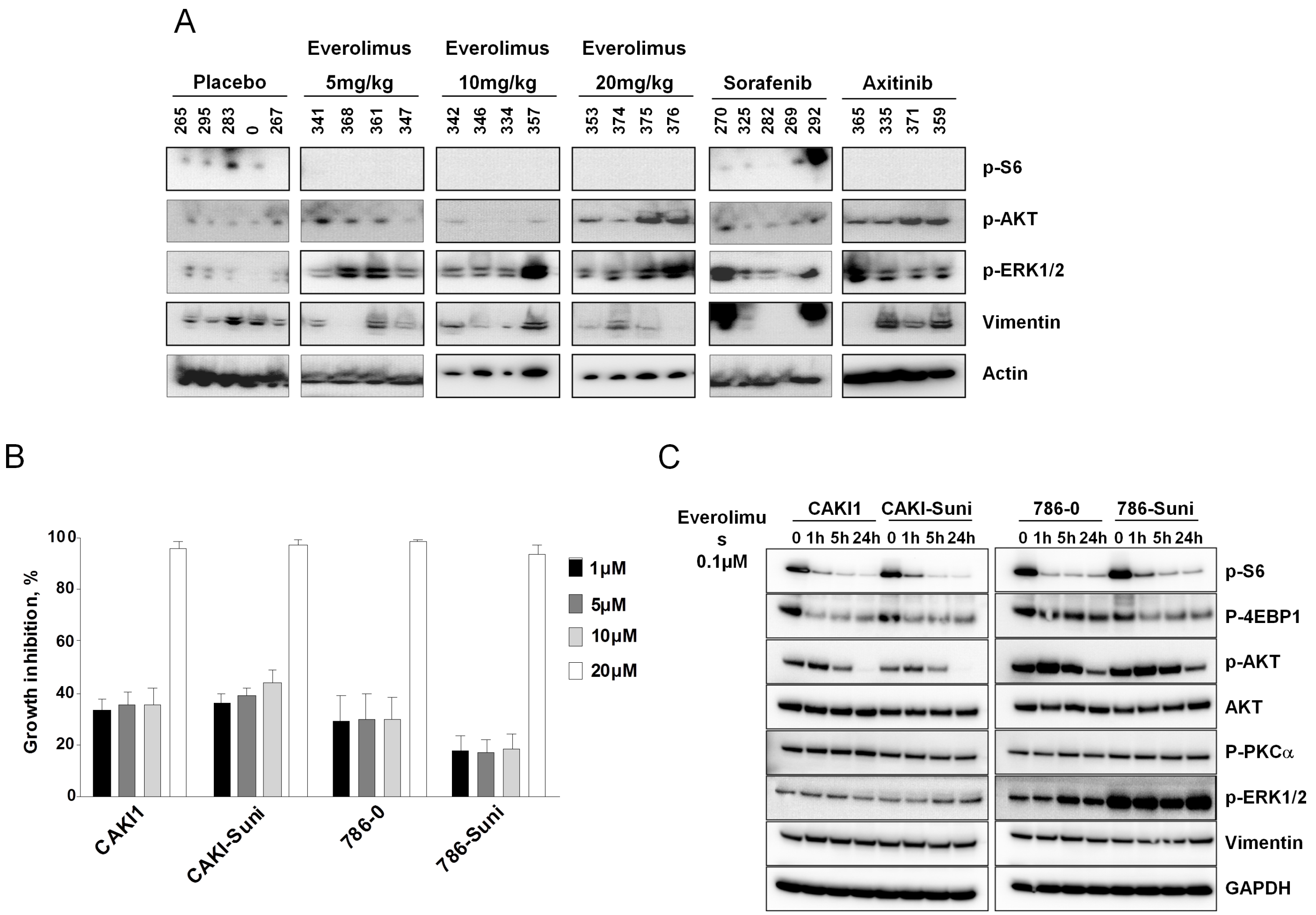

Supplementary Figure S4: ***In vivo*** **and** ***in vitro*** **effects of everolimus on sunitinib-resistant RCC models.** (**A**) Protein expression by western blot of p-S6, p-AKT, p-ERK1/2, Vimentin and Actin in sunitinib-resistant xenografts after second-line treatment with everolimus (5, 10, or 20 mg/kg), sorafenib (60 mg/kg), or axitinib (60 mg/kg). (**B**) Antiproliferative effects of 1, 5, 10 and 20 μM everolimus on CAKI1, CAKI-Suni, 786–0 and 786-Suni cell lines in vitro. Bars indicate the mean ± SEM of three independent experiments. (**C**) Protein expression by western blot of several genes in CAKI-1, CAKI-Suni, 786-0, and 786-Suni cells after 1-, 5-, and 24-hour exposure to 0.1 μM everolimus.

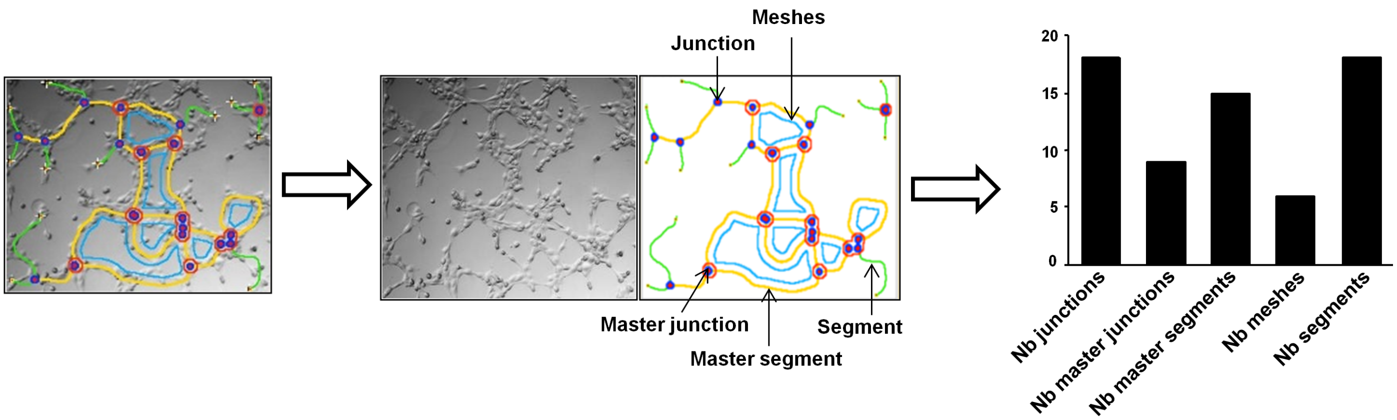

Supplementary Figure S5: Schematic representation of vasculogenic mimicry analysis *in vitro*.

Supplementary Table S1: Clinical & pathological characteristics of patients

**Clinical & pathological characteristics**	***N* = 24**
**Age** (median)	**55 years** (range: 45–80)
**Gender**	
Female	**3**
Male	**21**
**Tumor type**	
Clear cell RCC	**24**
Grade 2/3/4	**5/15/4**
**Initial treatment**	*24*
Nephrectomy	**22**
Sunitinib neo-adj. followed by nephrectomy	**1**
Radiotherapy followed by nephrectomy	**1**
Peri-operative Sunitinib	**3**
**Delays between diagnosis and sunitinib treatment**	median = **1.5 years** (range: 0–12)
< 1 year	**10**
1–3 years	**7**
> 3 years	**7**
**Treatment at relapse**	*24*
Sunitinib	**24**
**Following next line treatments** (excluding surgery)	
Sunitinib	**17**
INFa+sunitinib	**1**
Sorafenib	**2**
mTOR inhibitor	**9**

Supplementary Table S2: Tumoral CD31 staining in clear cell renal cell carcinoma patients

Patient #	Positive *versus* negative tumoral CD31 staining	Vessels associated with positive CD31 staining (%)
Pt11706	positive	20
Pt11608	negative	0
Pt13127	positive	80
Pt00451	positive	50
Pt03159	positive	60
Pt03201	negative	0
Pt03253	positive	5
Pt02907	positive	3
Pt05447	positive	40
Pt00713	positive	4
Pt16787	negative	0
Pt00413	negative	0
Pt16692	negative	0
Pt03530	positive	30
Pt10614	negative	0
Pt08374	positive	20
Pt16572	negative	0
Pt01127	positive	30
Pt01408	negative	0
Pt15761	negative	0
Pt07162	positive	10
Pt14472	negative	0
Pt14934	positive	40
Pt16909	negative	0

Supplementary Data File S1: Genes name and statistically significant expression changes between first line sunitinib (progressive or responders) and placebo treated tumors.

Supplementary Data File S2: Genes name and statistically significant expression changes between second line axitinib, sorafenib, everolimus, and placebo treated tumors.
